# RBM15 mediates m6A methylation modification of FOSB mRNA to promote dysfunction of trophoblast cells-a potential link to preeclampsia

**DOI:** 10.1186/s41065-025-00592-4

**Published:** 2025-11-21

**Authors:** Yan Li, Fei Li, Qian Gao

**Affiliations:** Department of Obstetrics and Gynaecology, Xiangyang No.1 People’s Hospital, No.50, Daqing Road, Fancheng District, Xiangyang City, Hubei 441000 China

**Keywords:** Preeclampsia, Trophoblast dysfunction, M6A methylation, RNA binding motif protein 15, FBJ murine osteosarcoma viral oncogene homolog b, YTH N6-methyladenosine RNA-binding protein 1

## Abstract

**Background:**

N6-methyladenosine (m6A) methylation modification plays an essential role in the molecular pathogenesis of preeclampsia (PE). This study aimed to explore m6A modification in in vitro PE model, involving RNA binding motif protein 15 (RBM15) and FBJ murine osteosarcoma viral oncogene homolog B (FOSB).

**Methods:**

PE was induced by lipopolysaccharide (LPS) in HTR8/SVneo cells. Real-time quantification PCR was used for mRNA detection and Western blotting was used for protein detection. Cell cytokines were examined using enzyme-linked immunosorbent assay. Cell functions were evaluated using EdU assay, flow cytometry, and transwell assay. RNA immunoprecipitation assay and dual-luciferase reporter assay were utilized for validating molecular interaction.

**Results:**

FOSB was highly expressed in placenta tissues from PE patients. Functionally, LPS-induced inflammation, proliferation inhibition, cell apoptosis and migration suppression were significantly abolished following FOSB knockdown. RBM15 upregulated FOSB expression via inducing m6A modification of FOSB mRNA. YTH N6-methyladenosine RNA-binding protein 1 (YTHDF1) acted as an m6A reader protein, and RBM15 enhanced the binding between YTHDF1 and FOSB. RBM15 knockdown relieved LPS-caused trophoblast cell injury by inhibiting FOSB.

**Conclusion:**

These results collectively suggested that RBM15 accelerated trophoblast cell dysfunction via mediating m6A modification of FOSB mRNA through the identification by YTHDF1.

**Supplementary Information:**

The online version contains supplementary material available at 10.1186/s41065-025-00592-4.

## Introduction

Preeclampsia (PE) is among the most severe complications of pregnancy, accompanied by increased maternal and perinatal morbidity and mortality rates [1]. Moreover, pregnant women with a history of PE face an elevated risk of cardiovascular and neurological diseases [2]. Placenta is vital to fetal growth and development during pregnancy, and placental abnormalities are thought to be a potential cause of various pregnancy complications, including PE [3]. PE is considered to be characterized by impaired uteroplacental perfusion followed by vascular inflammation and endothelial dysfunction [4]. Abnormal differentiation of trophoblast cells, the epithelial cells in the placenta, leads to placental disorder and the occurrence of PE [5]. Trophoblast stem cells can differentiate into multiple trophoblast subtypes, including proliferating mononuclear cytotrophoblasts (CTBs), multinucleated syncytiotrophoblast (STB), and extravillous trophoblast (EVT) cells with “directed invasion” ability to the maternal decidua [6]. The normal functions of trophoblast cells such as proliferation and migration are preconditions of successful pregnancy [7]. Thus, investigating the molecular mechanism underlying trophoblast cell dysfunction is crucial for understanding the pathogenesis of PE.

N6-methyladenosine (m6A), the most prevalent and conserved modification, exhibits essential implication in the physiological and pathological progression of human diseases. In m6A modification, RNAs can be reversibly and dynamically modified by methyltransferases (writers), demethylases (erasers) and binding proteins (readers) [8]. Current research progress has reported that m6A modification is linked to trophoblast cell behaviors, including proliferation, apoptosis, migration and invasion [9]. Exploration of m6A modification is possible to provide biological targets for the diagnosis and treatment of PE. RNA binding motif protein 15 (RBM15), an m6A methyltransferase, can contribute to oxidative stress and ferroptosis of trophoblast cells [10]. FBJ murine osteosarcoma viral oncogene homolog B (FOSB) is a subunit of activated protein 1 (AP-1), and AP-1 activity is related to the reduced ability of trophoblast to invasion [11]. A recent study has indicated that RBM15 induced m6A modification of 5-hydroxytryptamine receptor 1D (HTR1D) in regulating trophoblast cells [12]. Whether RBM15 can mediate m6A modification of FOSB remains fully unclear.

As a well-known m6A reader protein, YTH N6-methyladenosine RNA-binding protein 1 (YTHDF1) plays a pivotal role in enhancing mRNA stability or promoting protein translation [13]. YTHDF1 has been associated with pathological diseases such as inflammatory diseases and cancers [14]. Therefore, this study proposed a hypothesis that RBM15 may mediate FOSB m6A modification in PE and YTHDF1 can serve as a reader protein for FOSB m6A modification. The effects of RBM15/FOSB axis on trophoblast cells were explored, with the aim of clarifying a novel regulatory mechanism in PE compared to existing literature.

## Materials and methods

### Placenta specimens

A total of 28 severe PE patients (early-onset: *n* = 8, late-onset: *n* = 20) and 28 normal pregnant women at Xiangyang No.1 People’s Hospital, Hubei University of Medicine were enrolled in this study, based on acquisition of the informed consent from all individuals. After delivery, the placenta specimens were immediately collected from the maternal side of the placenta, with tissues showing hemorrhage, infarction or calcification excluded. The protocols were authorized by the Ethics Committee of Xiangyang No.1 People’s Hospital, Hubei University of Medicine. These obtained samples were washed with phosphate buffer solution (PBS; Gibco, Carlsbad, CA, USA) and then stored in liquid nitrogen.

### Lipopolysaccharide (LPS) treatment

Human trophoblast cell line HTR8/SVneo (BeNa, Xinyang, China) were incubated with RPMI-1640 medium (Gibco) mixing with 10% fetal bovine serum (Gibco), and cultured in the constant environment (temperature: 37℃, atmosphere: 95% air and 5% CO_2_). To construct the in vitro PE model, HTR8/SVneo cells were stimulated with 100 ng/mL LPS (Sigma, St. Louis, MO, USA) for 24 h to trigger a trophoblastic inflammatory response, referring to this concentration as previously described [15].

### Stable transfection of cells

The short hairpin RNA (shRNA) expression vectors shFOSB-1/shFOSB-2, shRBM15, and shYTHDF1 from GenePharma (Shanghai, China) were used for expression knockdown, and the no-targeting vector (shNC) acted as the control group. In addition, overexpression vectors (oeRBM15, oeYTHDF1, and oeFOSB) and the normal control vector (oeNC) were acquired from RIBOBIO (Guangzhou, China). 70% confluent HTR8/SVneo cells were transfected with the above vectors using Lipofectamine™ 3000 reagent, according to the specification from Invitrogen (Carlsbad, CA, USA).

### Real-time quantitative PCR (RT-qPCR) for mRNA expression

For total RNA extraction, specimens and cells were lysed with Trizol reagent (KeyGEN, Nanjing, China). Through the application of One-step RT-qPCR Kit (SYBR Green) (KeyGEN), reverse transcription and real-time PCR were administrated. All used primers were revealed in Table 1. Data analysis was implemented via the 2^-∆∆Ct^ method, with the expression standardization by an endogenous control β-actin.

**Table 1:** Primer sequences used for RT-qPCR

Name	Primers for PCR (5’−3’)	
FOSB	Forward	GCGCCGGGAACGAAATAAACT
	Reverse	CAAATCTCTCACCTCCGCCAG
RBM15	Forward	AAGGCACTGGCCAAATCTGA
	Reverse	ACTATAACAGGGTCAGCGCC
β-actin	Forward	CTTCGCGGGCGACGAT
	Reverse	CCACATAGGAATCCTTCTGACC

### Western blotting (WB) for protein level

Radioimmunoprecipitation assay (RIPA) buffer (KeyGEN) was utilized to isolate proteins from human samples and HTR8/SVneo cells. Proteins were loaded on gels through sodium dodecyl sulfate polyacrylamide gel electrophoresis (SDS-PAGE), and then instantly moved to polyvinylidene fluoride (PVDF) membranes (KeyGEN). Non-specific protein signals were prevented by incubating the membranes in 1% Blocking Solution (KeyGEN). Then, the primary antibody targeting FOSB (Invitrogen, MA1-41121), RBM15 (Proteintech, Wuhan, China; 66059−1-Ig), YTHDF1 (Proteintech, 66745-1-Ig) and β-actin (Proteintech, 66009-1-Ig) were incubated overnight at 4℃. The protein blots on the membranes were shown by ECL detection kit (KeyGEN) after the binding of the primary antibody with the secondary antibody (Proteintech, SA00001-1).

### Enzyme-linked immunosorbent assay (ELISA) for inflammation

The harvested HTR8/SVneo cells were used for ELISA detection. Levels (pg/mL) of interleukin-6 (IL-6), tumor necrosis factor-α (TNF-α), IL-1β, and soluble Fms-like tyrosine kinase-1 (sFlt-1) were examined through Human IL-6/TNF-α/IL-1β ELISA Kits (KeyGEN) and Human sFlt-1 ELISA kit (Amylet Scientific, Wuhan, China). The operation steps were in strict line with the users’ guidelines.

### Ethynyl-2’-deoxyuridine (EdU) assay for proliferation

Assessment of proliferation was performed via EdU Detection Kit (KeyGEN). HTR8/SVneo cells were fixed in 4% neutral paraformaldehyde, permeabilized with 0.5% Triton X-100 solution and incubated with Click-iT EdU reaction buffer. Following DNA staining by 4,6-Diaminooxazole-2-phenylindole (DAPI), cells were determined under the fluorescence microscope (Olympus, Tokyo, Japan). Cells with overlapped EdU and DAPI signals (EdU-positive cells) were defined as proliferating cells.

### Flow cytometry for apoptosis

Flow cytometry was carried out to evaluate apoptosis using Annexin V-FITC/PI Apoptosis Kit (KeyGEN). In accordance with the instruction book, 4 × 10^5^ HTR8/SVneo cells were firstly mixed with 5 µL Annexin V-fluorescein isothiocyanate (Annexin V-FITC) and then added with 5 µL propidium iodide (PI). After reaction in the dark, cell analysis was performed using the flow cytometer (BD Biosciences, San Diego, CA, USA). Annexin V^+^/PI^−^ and Annexin V^+^/PI^+^ labeled cells were recorded as apoptotic cells.

### Transwell assay for migration

1 × 10^4^ HTR8/SVneo cells suspended in serum-free medium were seeded into transwell chamber (Corning Inc., Corning, NY, USA), and the lower 24-well plate was filled with the complete medium. Then, migrated cells were fastened in 4% paraformaldehyde (Sigma) and stained in 0.1% crystal violet (Sigma). Cell images were obtained through an inverted microscope (Olympus) and cells were counted.

### RNA immunoprecipitation (RIP) assay

For RBM15 and FOSB, methylated RIP (MeRIP) was performed by m6A Methylation RIP Kit (RIBOBIO) after overexpression or knockdown of RBM15. Total RNA was fragmented and incubated with protein A/G magnetic beads containing anti-m6A or anti-IgG, then eluted RNA was utilized for FOSB mRNA detection through RT-qPCR. For YTHDF1 and FOSB, Magna RIP RNA-Binding Protein Immunoprecipitation Kit (Millipore, Billerica, MA, USA) was used following the manual. Lysates from HTR8/SVneo cells transfected with oeNC/oeRBM15 were incubated with anti-IgG or anti-YTHDF1 coated protein A/G magnetic beads followed by RNA isolation and FOSB mRNA enrichment detection.

### FOSB mRNA stability assay

Actinomycin D (Act D) can block the synthesis of new mRNA, enabling researchers to observe the degradation process of existing mRNA within cells. HTR8/SVneo cells with transfection of oeNC/oeRBM15 or oeNC/oeYTHDF1 were treated with 5 mg/mL Act D (Sigma) for 0 h, 4 h, or 8 h. At each time point, total RNA was isolated and FOSB mRNA was quantified using RT-qPCR.

### Dual-luciferase reporter assay

The m6A binding site (with the highest scoring for the predicted positions) in FOSB was mutated and cloned into the pGL4 vector (Promega, Beijing, China) to obtain mutant-type FOSB (MUT-FOSB), with wild-type FOSB (WT-FOSB) as the non-mutated control. HTR8/SVneo cells were co-transfected with WT-FOSB/MUT-FOSB and oeNC/oeRBM15/oeYTHDF1, followed by luciferase activity detection using the Dual-luciferase Reporter Assay System (Promega).

### Statistical analysis

Pearson’s correlation coefficient was applied for analysis of linear relationship between gene expression. Results were collected from three independent experiments and presented as the mean ± SD. SPSS and GraphPad Prism were utilized to perform the statistical analysis. Normal distribution was analyzed through Shapiro-Wilk test, and homogeneity of variance was analyzed via Levene test. All data conformed to normal distribution and homogeneity of variance, and thus parametric tests were selected. For comparison of group difference, Student’s *t*-test was used for two groups, while analysis of variance (ANOVA) was conducted for multiple groups. Statistical difference was significant with *P* < 0.05.

## Results

### PE patients expressed FOSB at a high level

**Fig. 1:**
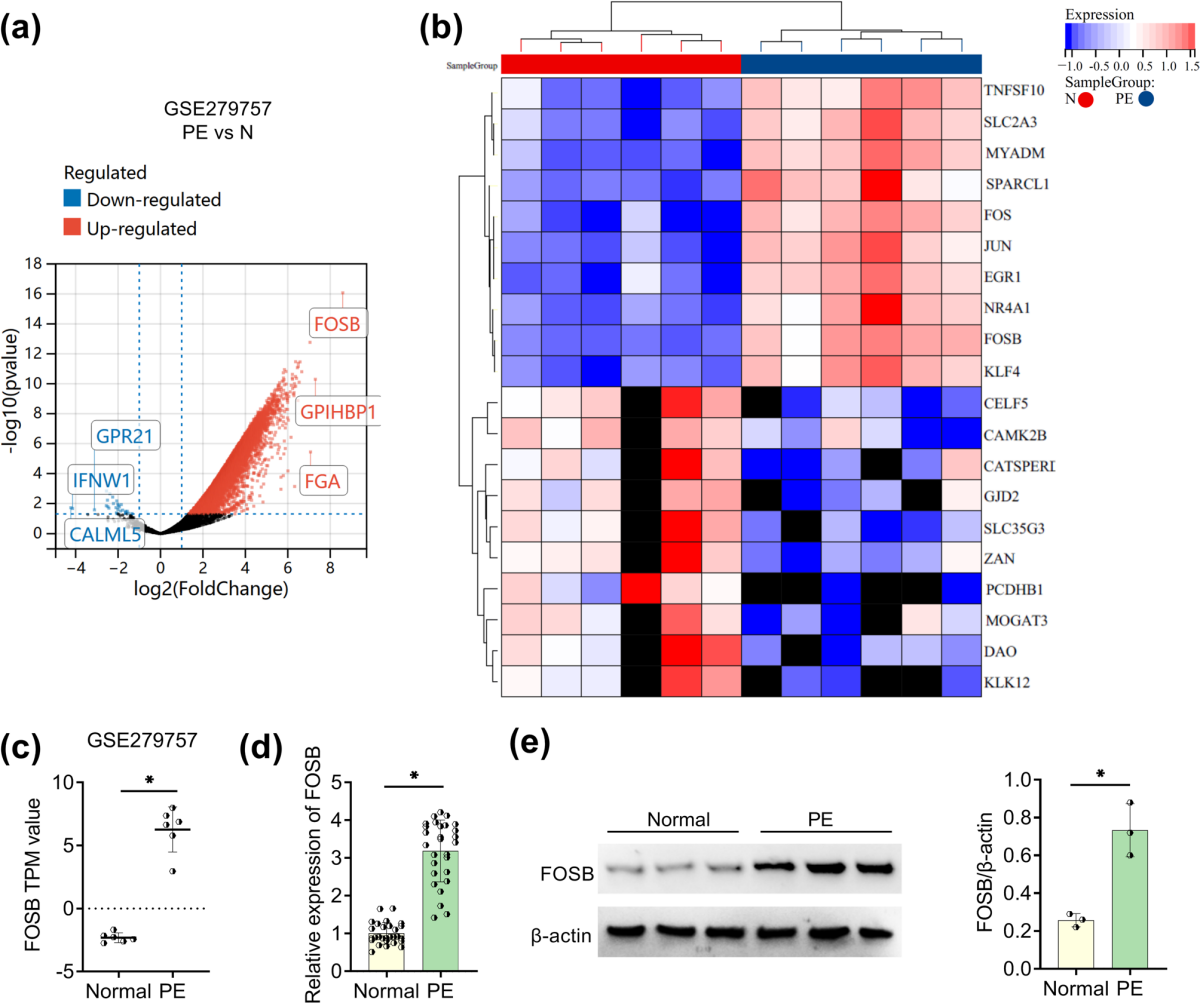
PE patients expressed FOSB at a high level. **a** Volcano plot of differential genes in PE samples versus normal samples from GSE279757 dataset. **b** Heat map showed the clustering analysis of top 10 differential genes. **c** FOSB TPM value in GSE279757 dataset. **d-e** RT-qPCR for FOSB mRNA detection (d, *n* = 28) and WB for FOSB protein examination (e, *n* = 3) in placenta tissues from PE patients and normal pregnant women. Each experiment was biologically repeated three times (*n* = 3). ^*^P < 0.05

GSE279757 dataset analysis was performed using Sangerbox website. The expression value in GSE279757 dataset was converted from FPKM to TPM through a RNA-seq conversion tool, and the differential expression genes between placenta tissues of PE patients and normal pregnant women were analyzed by limma quick difference analysis tool. The volcano plot in Fig. 1a showed the differential genes according to adj.P.Val < 0.05 [log2FC] > = 1. FOSB, GPIHBP1, and FGA were the top 3 up-regulated genes, while GPR21, IFNW1, and CALML5 were the top 3 down-regulated genes. Then, the heat map indicated the clustering analysis of the top 10 up/down-regulated genes from PE placental tissues in GSE279757 dataset (Fig. 1b). FOSB TPM value in PE group was conspicuously elevated contrasted to normal group in GSE279757 dataset (Fig. 1c). The basic characteristics of PE and normal groups were shown in Table 2, in which the PE group exhibited difference to the normal group in gestational age at delivery, blood pressure, urine protein, and neonatal weight. Subsequently, results from RT-qPCR and WB affirmed the high expression of FOSB both mRNA and protein in collected placental specimens from PE patients relative to normal controls (Fig. 1d and e). Indeed, FOSB was an up-regulated gene in PE.

**Table 2:** The basic characteristics of the study populations

	Normal (*n* = 28)	PE (*n* = 28)	*P* value
Maternal age (years)	30.6 ± 2.8	31.2 ± 3.9	0.51
Gestational age at delivery (weeks)	38.3 ± 1.7	34.2 ± 1.1	< 0.05
Body mass index (kg/m^2^)	24.2 ± 2.5	24.9 ± 2.2	0.27
Systolic blood pressure (mmHg)	121 ± 7.2	159 ± 8.8	< 0.05
Diastolic blood pressure (mmHg)	78 ± 5.1	101 ± 6.7	< 0.05
Smokers % (n)	7.1(2)	14.3(4)	0.67
Urine protein (g/24 h)	0.08 ± 0.02	3.24 ± 1.1	< 0.05
Neonatal weight (g)	3317 ± 402	2289 ± 523	< 0.05

### Inhibiting FOSB expression ameliorated LPS-induced inflammation reaction

**Fig. 2:**
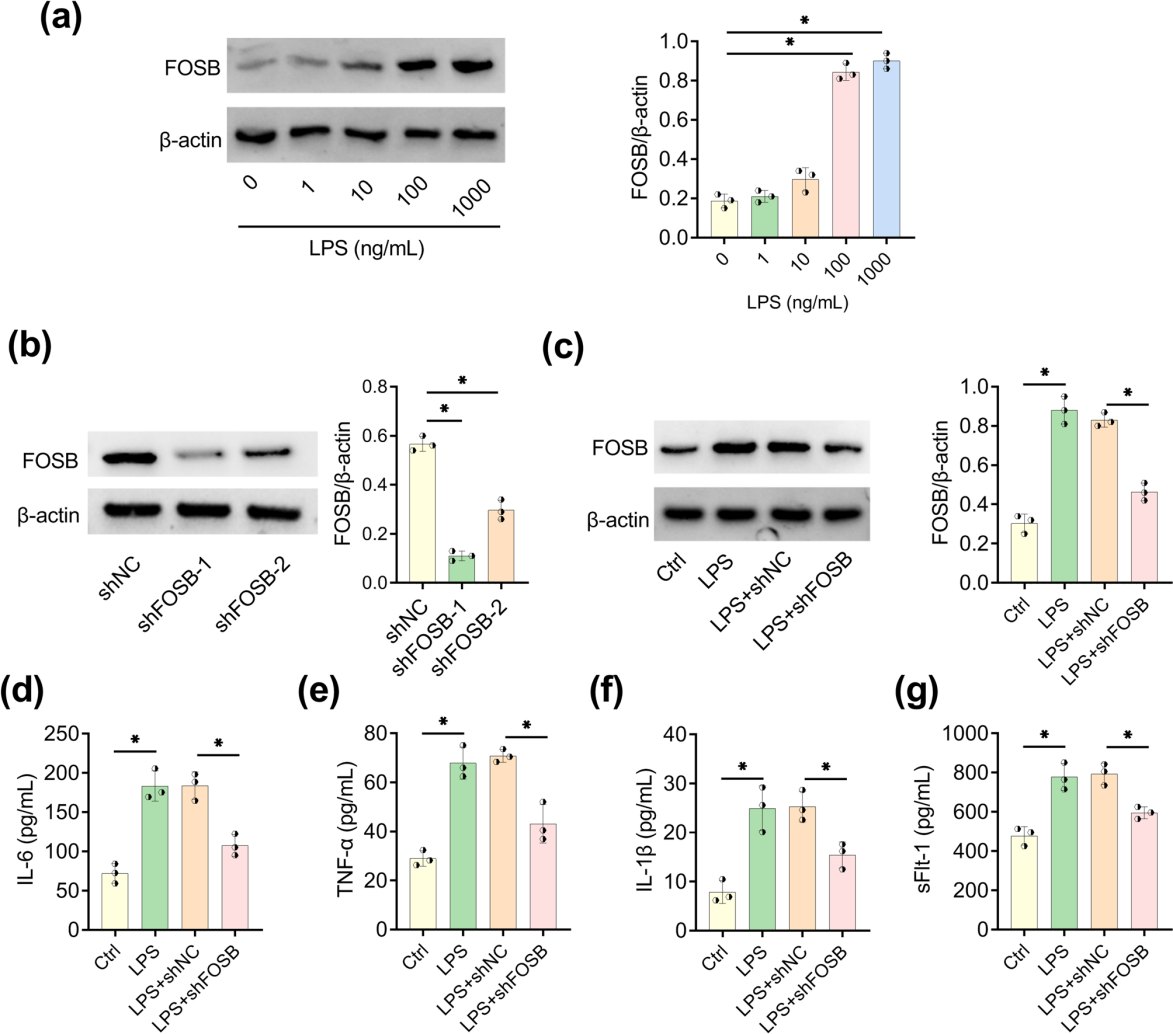
Inhibiting FOSB expression ameliorated LPS-induced inflammation reaction. **a** FOSB expression was measured by WB in HTR8/SVneo cells following treatment of LPS with 0 ng/mL, 1 ng/mL, 10 ng/mL, 100 ng/mL, and 1000 ng/mL for 24 h. **b** Transfection effects of shFOSB-1 and shFOSB-2 were detected using WB. **c** WB was employed to measure FOSB protein after HTR8/SVneo cells were treated with 100 ng/mL LPS and transfected with shNC/shFOSB. **d-g** ELISA was performed to examine the levels of IL-6 (d), TNF-α (e), IL-1β (f) and sFlt-1 (g). Each experiment was biologically repeated three times (*n* = 3). ^*^P < 0.05

FOSB protein level was changed by an obvious increase after LPS treatment with 100 ng/mL or 1000 ng/mL (Fig. 2a). Subsequent assays were carried out using 100 ng/mL as the treatment concentration of LPS. Transfection of shRNA vectors was used for FOSB knockdown, and FOSB protein downregulation was observed both in shFOSB-1 and sh-FOSB-2 groups (Fig. 2b). Due to the more evident inhibition of FOSB in shFOSB-1 group, shFOSB-1 was applied for further research and labeled as shFOSB. LPS resulted in a promoting effect on FOSB protein expression, and LPS + shFOSB group exhibited the reduction of FOSB protein contrasted to LPS + shNC group (Fig. 2c). Thus, knockdown efficiency of shFOSB was conspicuous in LPS-induced HTR8/SVneo cells. By performing ELISA, inflammatory cytokines IL-6/TNF-α/IL-1β (Fig. 2d, e, and f) and anti-angiogenic molecule sFlt-1 (Fig. 2g) promoted by LPS were all reduced with the introduction of shFOSB. These results suggested that FOSB knockdown inhibited LPS-induced inflammation in HTR8/SVneo cells.

### FOSB downregulation enhanced proliferation and migration but inhibited apoptosis of LPS-induced trophoblast cells

**Fig. 3:**
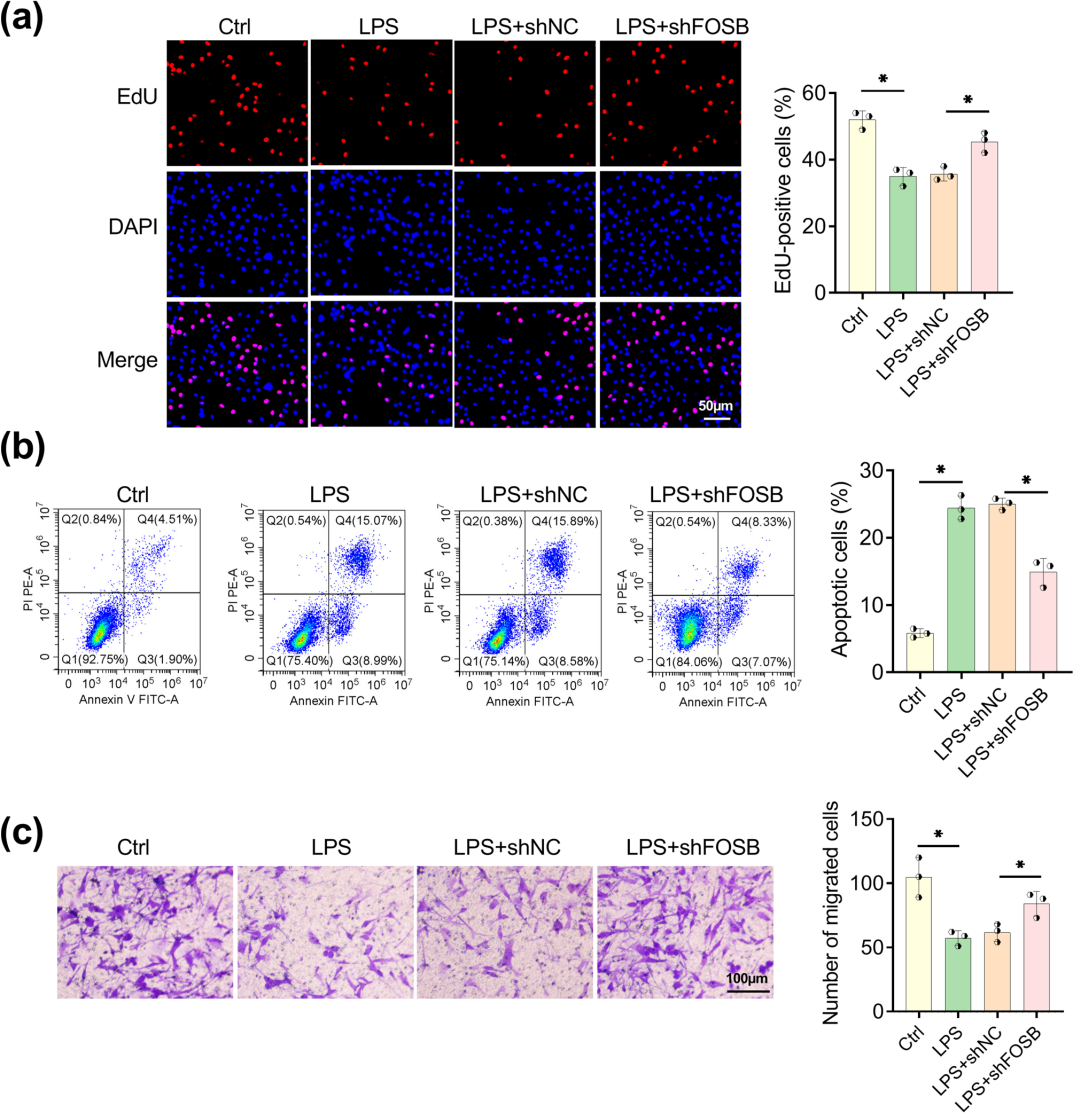
FOSB downregulation enhanced proliferation and migration but inhibited apoptosis of LPS-induced trophoblast cells. HTR8/SVneo cells after 100 ng/mL LPS treatment were transfected with shNC or shFOSB. **a** EdU assay was utilized to assess cell proliferation ability. **b** Flow cytometry was carried out to determine cell apoptosis. **c** Transwell assay was performed for examination of migrated cells. Each experiment was biologically repeated three times (*n* = 3). ^*^P < 0.05

In EdU assay, LPS decreased the number of EdU-positive cells (LPS group relative to control group) and then this influence was eliminated following the expression deficiency of FOSB (LPS + shFOSB group relative to LPS + shNC group) (Fig. 3a). Flow cytometry manifested that LPS enhanced cell apoptosis and the apoptotic cells were distinctly reduced in LPS + shFOSB group compared to LPS + shNC group (Fig. 3b). The migrated cells were reduced by LPS, whereas they were reversely increased in HTR8/SVneo after FOSB expression was knocked down (LPS + shFOSB group relative to LPS + shNC group) (Fig. 3c). FOSB inhibition could facilitate trophoblast cell proliferation and migration under LPS presence.

### RBM15 promoted FOSB mRNA expression via m6A modification

**Fig. 4:**
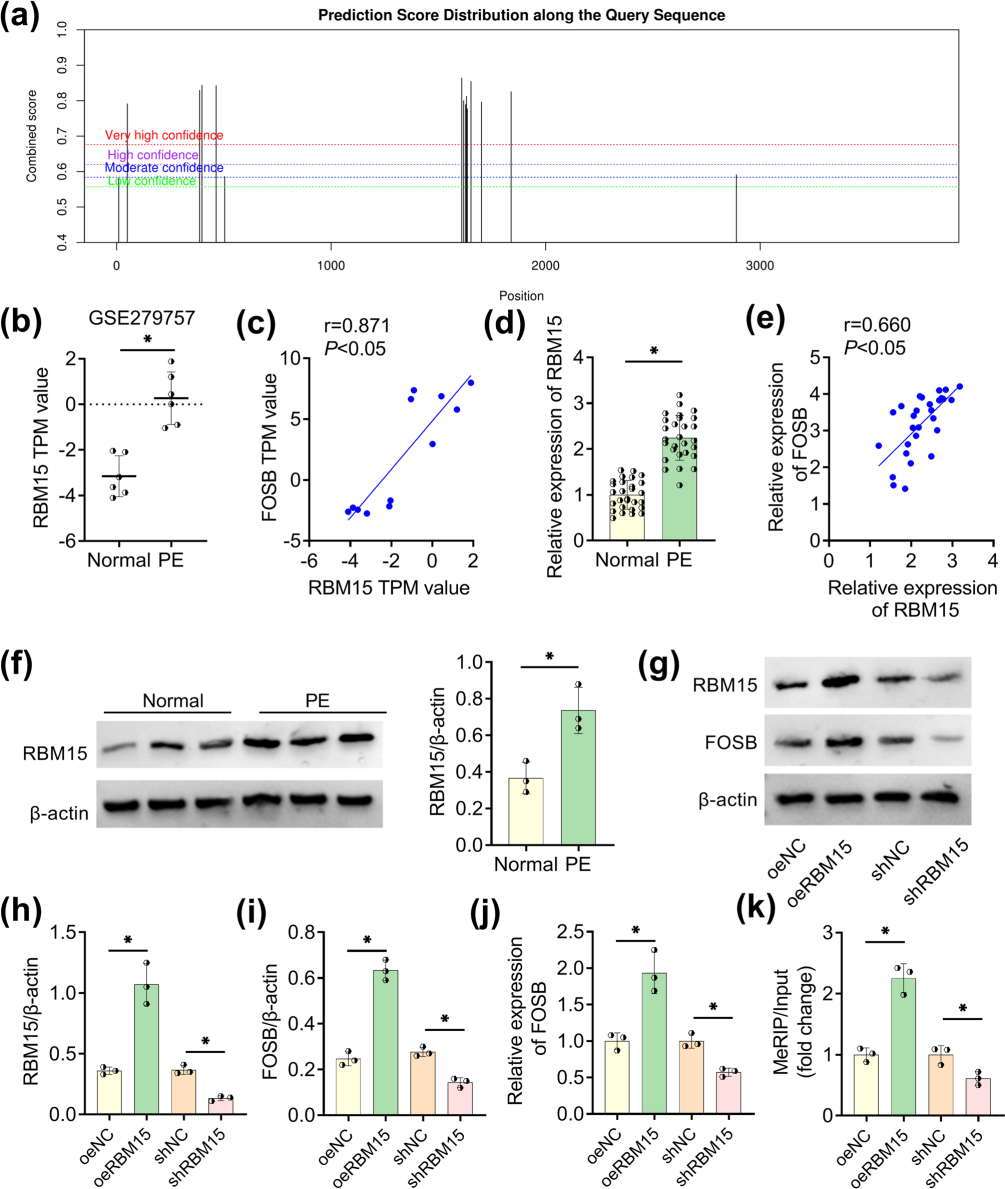
RBM15 promoted FOSB mRNA expression via m6A modification. **a** The m6A site in FOSB mRNA was predicted by SRAMP. **b-c** RBM15 expression in PE samples (b) and its linear association with FOSB in PE samples (c) according to GSE279757 dataset. **d** RT-qPCR for mRNA quantification of RBM15 in PE placenta tissues (*n* = 28) and normal controls (*n* = 28). **e **Pearson’s correlation coefficient was used for linear analysis between mRNA expression of RBM15 and FOSB. **f** RBM15 protein testing by WB in PE placenta tissues (*n* = 3) and normal placenta tissues (*n* = 3). **g-i** WB analysis (g) was conducted to determine protein levels of RBM15 (h) and FOSB (i) after HTR8/SVneo cells were transfected with oeNC/oeRBM15 or shNC/shRBM15. **j** FOSB mRNA expression was quantified via RT-qPCR following transfection of oeNC/oeRBM15 or shNC/shRBM15. **k** MeRIP was used for detection of m6A-modified FOSB mRNA level after overexpression or knockdown of RBM15. Each experiment was biologically repeated three times (*n* = 3). ^*^P < 0.05

The m6A binding site in FOSB mRNA was predicted through SRAMP (http://www.cuilab.cn/sramp) (Fig. 4a). RBM15 was found as a methylation-related enzyme with binding sites to FOSB mRNA in ENCORI database. In GSE279757 dataset, RBM15 TPM value was much higher in PE placenta specimens than that in normal placenta specimens (Fig. 4b). A positive correlation (*r* = 0.871, *P* < 0.05) between RBM15 and FOSB expression was found in PE tissues from GSE279757 dataset (Fig. 4c). Likewise, the aberrant upregulation of RBM15 mRNA was detected in placenta tissues of PE patients (Fig. 4d) and RBM15 was positively correlated to FOSB (*r* = 0.660, *P* < 0.05) in placenta tissues (Fig. 4e). WB further validated that RBM15 protein expression was elevated in PE patients relative to normal pregnant women (Fig. 4f). By performing WB analysis (Fig. 4g), RBM15 (Fig. 4h) and FOSB (Fig. 4i) protein levels were indicated to be increased by oeRBM15 transfection and suppressed by shRBM15 transfection when compared to control group oeNC or shNC, respectively. Furthermore, overexpression of RBM15 evoked the mRNA upregulation of FOSB and knockdown of RBM15 led to a reverse effect on FOSB mRNA (Fig. 4j). MeRIP suggested that m6A-modified FOSB mRNA level was promoted in oeRBM15 group and restrained in shRBM15 group, relative to the respective control group (Fig. 4k). Hence, RBM15 could up-regulate FOSB expression through the m6A modification of FOSB mRNA.

### RBM15 enhanced FOSB mRNA stability via YTHDF1

**Fig. 5:**
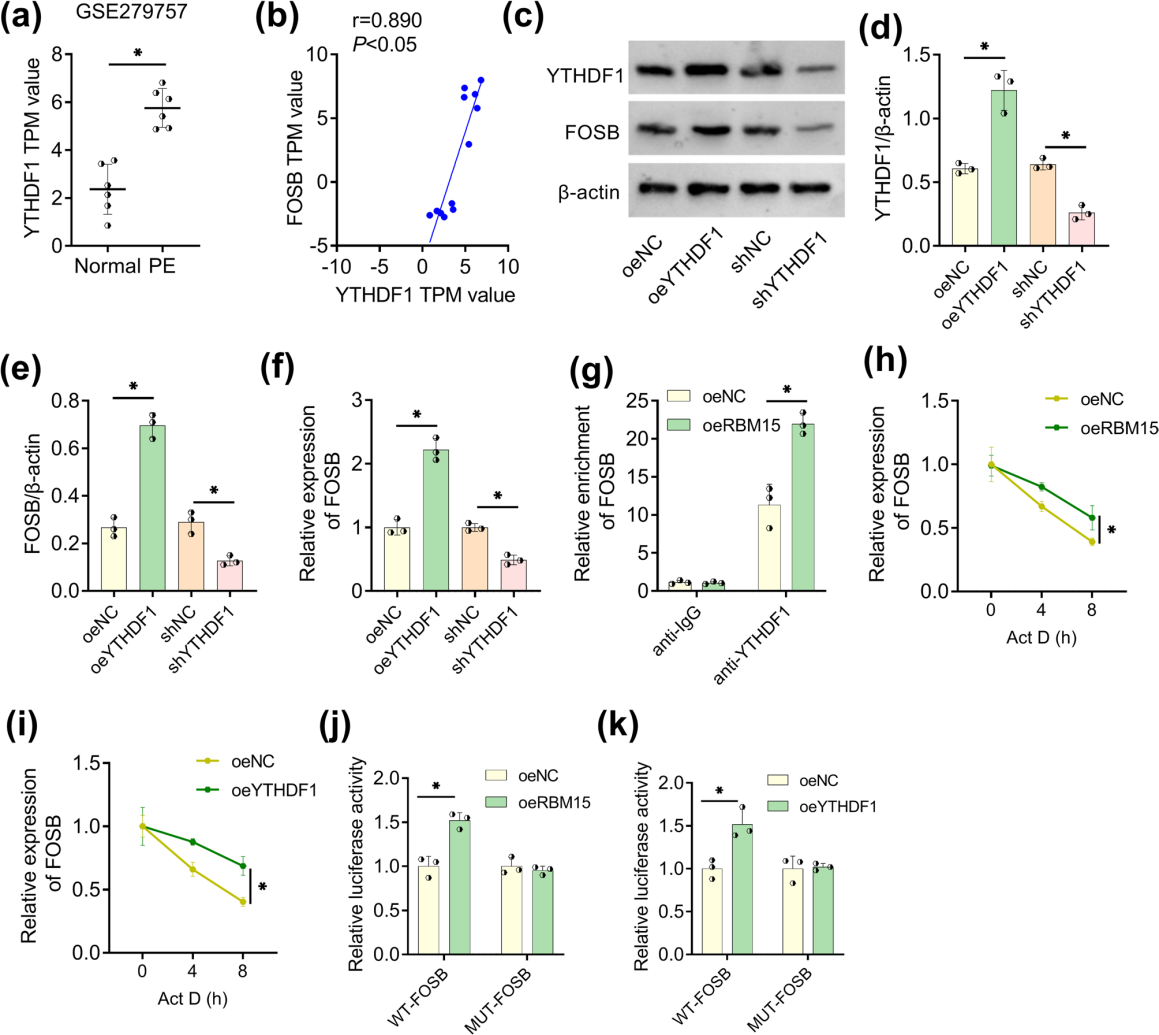
RBM15 enhanced FOSB mRNA stability via YTHDF1. **a-b** YTHDF1 expression in PE placenta tissues (a) and the linear relationship between YTHDF1 and FOSB (b) in GSE279757 dataset. **c-e** WB results (c) for YTHDF1 (d) and FOSB (e) in oeNC/oeYTHDF1 or shNC/shYTHDF1 transfected HTR8/SVneo cells. **f** RT-qPCR was applied for analyzing the effects of YTHDF1 upregulation and downregulation on FOSB mRNA. **g** The influence of RBM15 on the interaction between YTHDF1 and FOSB was assessed using RIP assay. **h-i** FOSB mRNA stability was measured via Act D treatment and RT-qPCR in RBM15-overexpressed or YTHDF1-overexpressed HTR8/SVneo cells. **j-k** Dual-luciferase reporter assay was conducted to affirm the interaction between RBM15 or YTHDF1 and FOSB after mutagenesis. Each experiment was biologically repeated three times (*n* = 3). ^*^P < 0.05

According to the GSE279757 dataset, YTHDF1 was discovered to be up-regulated in PE samples contrasted to normal samples (Fig. 5a) and it was correlated to FOSB with a positive relationship (*r* = 0.890, *P* < 0.05) in PE placenta tissues (Fig. 5b). As the protein blots displayed in Fig. 5c, oeYTHDF1 group induced the facilitating regulation of YTHDF1 and FOSB compared with oeNC group, while shYTHDF1 group expressed lower YTHDF1 and FOSB versus shNC group (Fig. 5d and e). RT-qPCR data after YTHDF1 overexpression or inhibition showed that YTHDF1 could regulate FOSB mRNA level positively (Fig. 5f). RIP analysis suggested that FOSB was significantly enriched by anti-YTHDF1 relative to anti-IgG, and RBM15 upregulation contributed to the binding between YTHDF1 and FOSB (Fig. 5g). Under the mRNA synthesis inhibition by Act D, FOSB mRNA stability was observed to be enhanced with the expression increase of RBM15 (Fig. 5h) or YTHDF1 (Fig. 5i). In addition, dual-luciferase reporter assay revealed that RBM15 or YTHDF1 overexpression promoted relative luciferase activity of WT-FOSB rather than MUT-FOSB, suggesting the interaction between RBM15 or YTHDF1 with m6A binding site of FOSB (Fig. 5j and k). These consequences implied that RBM15 enhanced FOSB mRNA stability and promoted the identification of YTHDF1 for FOSB.

### RBM15 knockdown relieved LPS-evoked inflammation by inhibiting FOSB

**Fig. 6:**
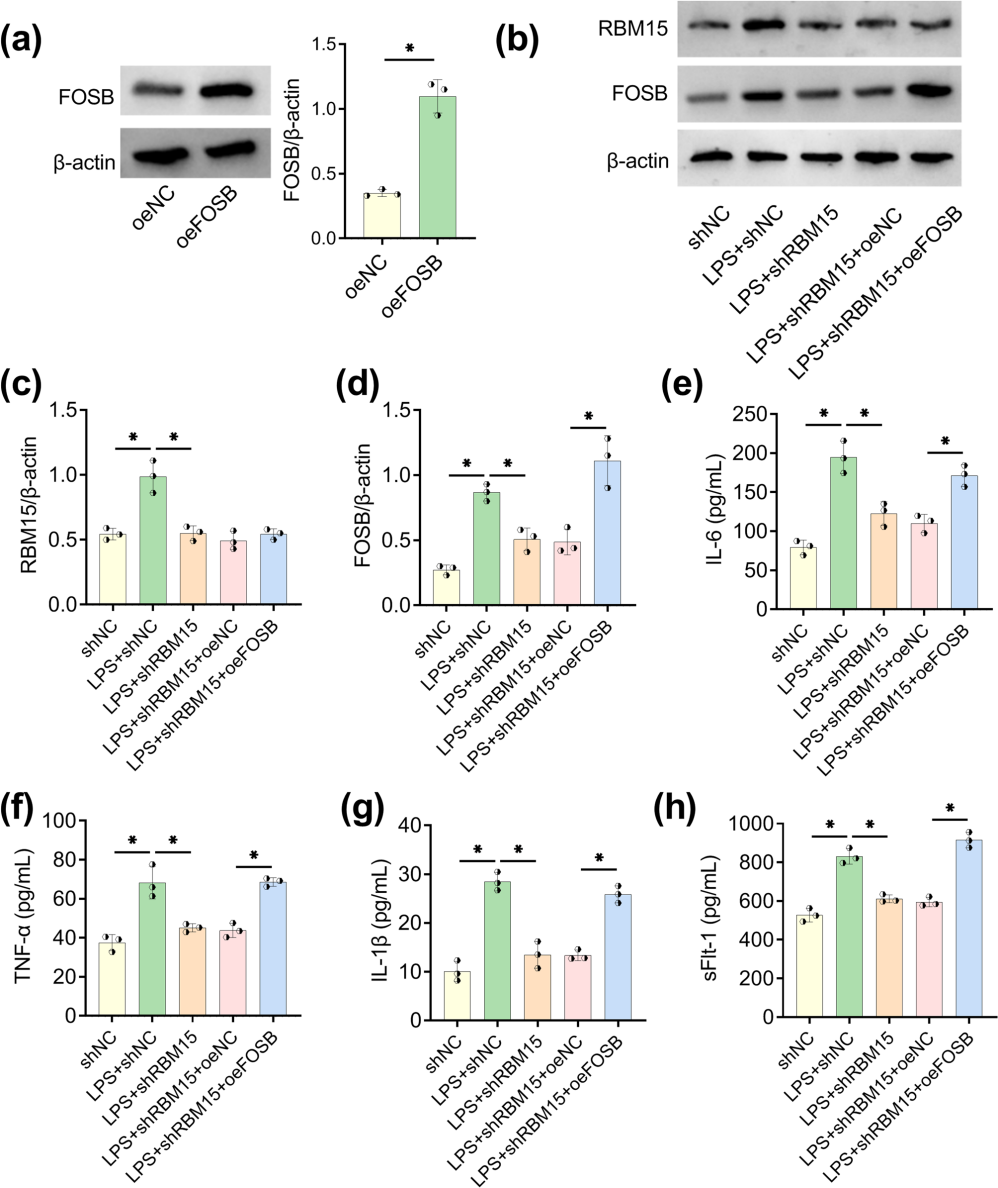
RBM15 knockdown relieved LPS-evoked inflammation by inhibiting FOSB. **a** Overexpression efficiency of oeFOSB was evaluated via WB. **b-h** HTR8/SVneo cells were treated with shNC, 100 ng/mL LPS + shNC, LPS + shRBM15, LPS + shRBM15 + oeNC, or LPS + shRBM15 + oeFOSB. (b-d) WB detection (b) was implemented for protein analysis of RBM15 (c) and FOSB (d). (e-h) IL-6 (e), TNF-α (f), IL-1β (g) and sFlt-1 (h) were examined via ELISA. Each experiment was biologically repeated three times (*n* = 3). ^*^P < 0.05

Introduction of oeFOSB markedly elevated FOSB protein expression relative to oeNC transfection (Fig. 6a). After WB detection (Fig. 6b), it was noticed that LPS-induced upregulation of RBM15 and FOSB was repressed by shRBM15. However, overexpression of FOSB did not affect RBM15 protein expression in LPS + shRBM15 group but it rescued the FOSB protein inhibition in LPS + shRBM15 group (Fig. 6c and d). Knockdown of RBM15 inhibited the production of IL-6/TNF-α/IL-1β (Fig. 6e, f, and g) and sFlt-1 (Fig. 6h) in LPS-treated HTR8/SVneo cells, which was subsequently counteracted by oeFOSB. Inflammation reaction caused by LPS was abated after the repression of RBM15/FOSB regulatory axis.

### RBM15/FOSB axis regulated LPS-mediated proliferation, apoptosis and migration

**Fig. 7:**
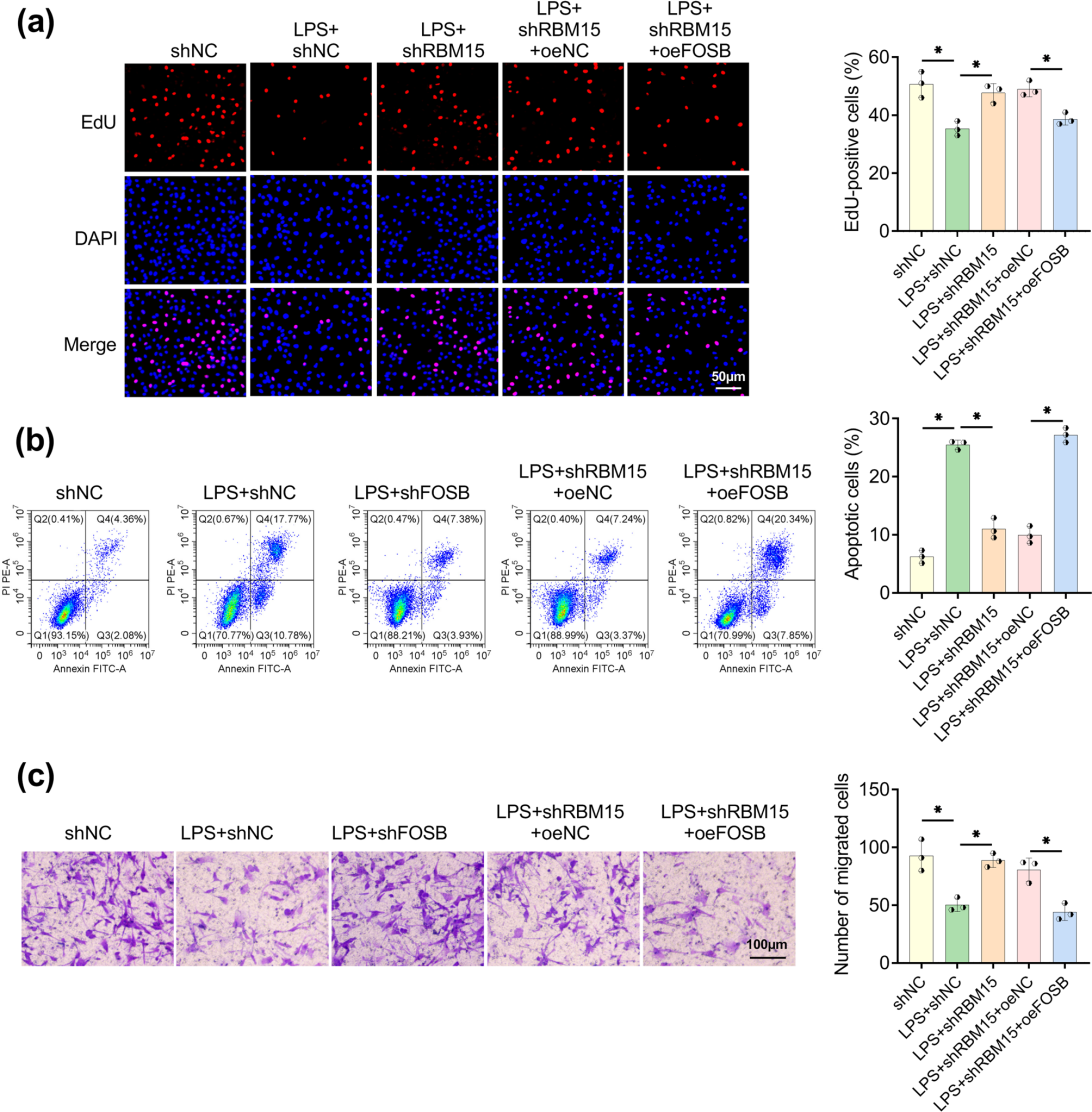
RBM15/FOSB axis regulated LPS-induced proliferation, apoptosis and migration. 100 ng/mL LPS-treated HTR8/SVneo cells were transfected with shNC, shRBM15, shRBM15 + oeNC, or shRBM15 + oeFOSB. **a** The proliferation was assessed using EdU assay. **b** Apoptotic cells were detected via flow cytometry. **c** Cell migration was evaluated through transwell assay. Each experiment was biologically repeated three times (*n* = 3). ^*^P < 0.05

EdU assay demonstrated that cell proliferation ability was accelerated in RBM15-knockdown HTR8/SVneo cells under LPS treatment, whereas overexpression of FOSB abrogated this proliferation promotion (Fig. 7a). Similarly, LPS-induced cell apoptosis was attenuated by shRBM15, and FOSB upregulation expedited apoptosis in LPS + shRBM15 co-treated HTR8/SVneo cells (Fig. 7b). The stimulative influence of RBM15 knockdown on cell migration following LPS induction was significantly abolished with the elevated FOSB expression (Fig. 7c). Altogether, RBM15 contributed to LPS-induced trophoblast cell dysfunction via upregulating FOSB.

## Discussion

Increasing evidence has highlighted the significance of understanding the molecular mechanism and discovering the predictive biomarkers in PE [16]. Herein, this study found the role of m6A-related signal axis RBM15/YTHDF1/FOSB in trophoblast cell dysfunction, indicating the potential involvement of m6A methylation modification in PE progression.

Online dataset analysis and expression detection in collected samples demonstrated the abnormal upregulation of FOSB in PE, which implicated the important function of FOSB in PE. Inadequate resolution of inflammatory response may contribute to the development of PE. Impaired invasion or remodeled arteries can develop hypoxia/reperfusion to result in oxidative stress in the placenta, which further increases pro-inflammatory cytokine secretion to trigger inflammatory response and facilitate PE progression [17]. In addition, poor placentation can elicit the release of anti-angiogenic factors like sFlt-1, inducing systemic inflammation and widespread endothelial dysfunction [18]. Through ELISA analysis for inflammation and sFlt-1, it was found that LPS-induced synthesis of inflammatory cytokines and inhibition of angiogenesis were restrained by FOSB knockdown. A previous study has unraveled that FOSB was up-regulated in endothelial cells exposed to high glucose and hypoxia, and it might be involved in inflammation and oxidative stress in vascular cell injury [19]. FOSB could mediate lung inflammation in shock-induced blast lung injury of rats [20]. Consistently, FOSB also exhibited pro-inflammatory function of trophoblast cells, suggesting its promoting regulation in PE.

FOSB has influenced biological functions of various cancer cells, including apoptosis, migration and proliferation. For example, in papillary thyroid cancer, FOSB was validated to play an oncogenic role with promoting modulation of cell growth, migration and invasiveness [21]. On the contrary, overexpression of FOSB has repressed cell proliferation and migration in gastric cancer [22]. However, its roles in trophoblast cell growth and migration are unclear. Trophoblast proliferation and migration are necessary for the growth of the placenta [23], and thus it is of great importance to perform functional exploration for FOSB. According to the current results, it was observed that FOSB downregulation recovered the normal proliferation and migration but blocked apoptosis of trophoblast cells. A previous study has indicated that FOS family has different effects on trophoblast cells. FOS knockdown can induce migration and invasion, while FOS1 knockdown exerts opposing regulation [24]. Herein, FOSB induced trophoblast dysfunction to possibly expedite the development of PE.

As one of the most abundant modification types of RNAs, m6A has attracted increasing attention in PE research. Gu et al.. reported that methyltransferase like 3 (METTL3) enhanced m6A RNA methylation of heterogeneous nuclear ribonucleoprotein C1/C2 (hnRNPC1/C2) to promote placental trophoblast disorder in PE [25]. Another PE study disclosed that Wilms’ tumor 1-associating protein (WTAP) improved high mobility group nucleosomal binding domain 3 (HMGN3) mRNA stability via m6A recognition protein insulin-like growth factor 2 mRNA-binding protein 1 (IGF2BP1) [26]. Given the m6A binding site in FOSB, this study speculated that FOSB was associated with m6A modification and discovered RBM15 had the potential interaction with FOSB mRNA. The further evidence revealed that RBM15 increased m6A-modified FOSB mRNA expression and FOSB mRNA stability, consistent with m6A modification of METTL3 and WTAP in PE [25, 26]. To explore the m6A reader protein responsible for FOSB m6A modification, YTHDF1 was discovered to increase FOSB mRNA stability and RBM15 could reinforce the YTHDF1/FOSB binding. RBM15 has been manifested to mediate m6A modification of transmembrane BAX inhibitor motif containing 6 (TMBIM6) mRNA via depending on the reader protein IGF2BP3 in laryngeal squamous cell carcinoma [27]. In hepatocellular carcinoma, m6A methylation of vascular endothelial growth factor A (VEGFA) mRNA was modified by methyltransferase RBM15 and two readers, YTHDF2 and IGF2BP3 [28]. Also, RBM15 mediated HTR1D m6A modification with YTHDF2 as a reader protein [12]. In conformity with these findings, the present results testified that FOSB mRNA was recognized by m6A reader YTHDF1 and up-regulated by RBM15 in an m6A-dependent way. Moreover, functional assays further confirmed that RBM15 promoted inflammation and apoptosis but impeded proliferation and migration of trophoblast cells by promoting FOSB. The effects of RBM15 in trophoblast cells were in line with that in a recent study [12]. Different from RBM15/YTHDF2/HTR1D, this study unraveled RBM15/YTHDF1/FOSB as a specific mechanism underlying RBM15-mediated m6A modification. Propofol has been found to facilitate trophoblast growth and migration by targeting METTL3-mediated m6A methylation [29]. Thus, clinical translation may be achieved by seeking possible agents (e.g., molecular drugs or traditional Chinese medicines) or using specific siRNA inhibitors targeting RBM15-mediated m6A modification.

There are still some potential limitations in the current condition. Firstly, LPS is used to induce pro-inflammatory phenotype in HTR-8/SVneo cells in not specific enough, although LPS has been widely used for establishment of in vitro PE model. In the future, the in vitro PE model will be improved to better promote PE research and support the current conclusion. Secondly, this study mainly focused on in vitro experiments, but in vivo validation is lacked due to experimental stage and cost. Further study on in vivo validation remains to be explored, providing more evidence for RBM15/YTHDF1/FOSB axis in PE.

To summarize, this report discovered the critical role of RBM15 in LPS-induced trophoblast dysfunction. Mechanistically, RBM15 recruited YTHDF1 to elevate FOSB mRNA stability, consequently contributing to trophoblast dysfunction. These findings revealed a novel perspective associated with m6A modification in PE pathogenesis, and provided the certain support for RBM15 or FOSB as the biological target for clinical management of PE.

## Supplementary Information

Supplementary Material 1.

Supplementary Material 2.

## Data Availability

No datasets were generated or analysed during the current study.
