# Supplementary figures and images for: RBM15 mediates m6A methylation modification of FOSB mRNA to promote dysfunction of trophoblast cells-a potential link to preeclampsia
Source: Hereditas. 2025 Nov 21;162:241. doi: 10.1186/s41065-025-00592-4 (PMC12752175; doi:10.1186/s41065-025-00592-4)

Fig 1e

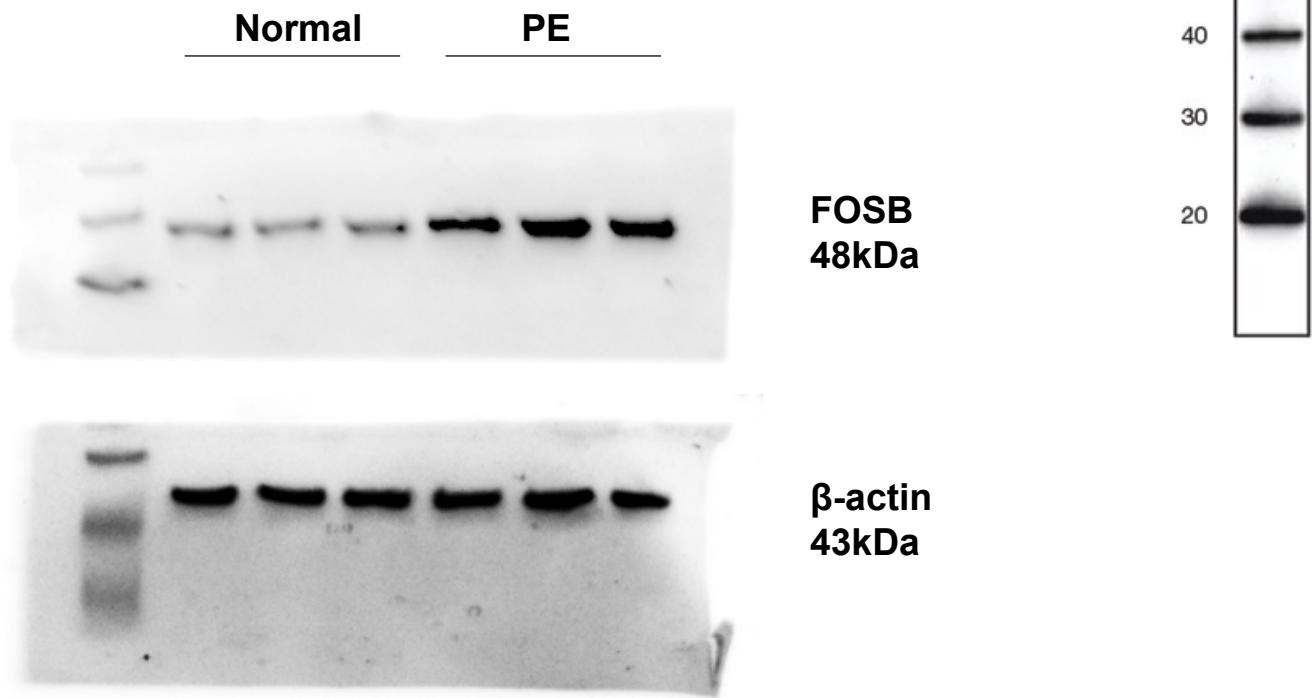

**Fig 2a**

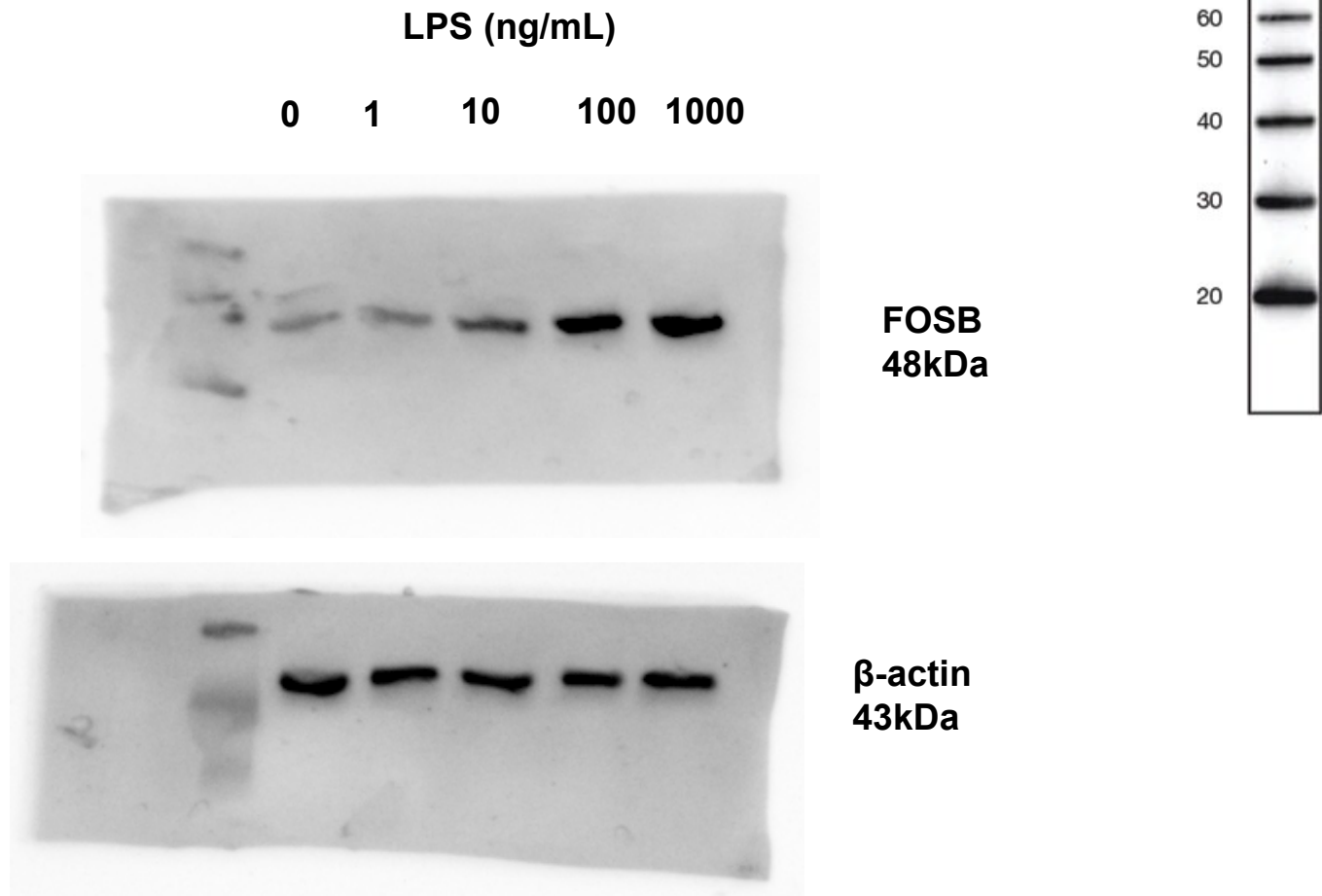

Fig 2b

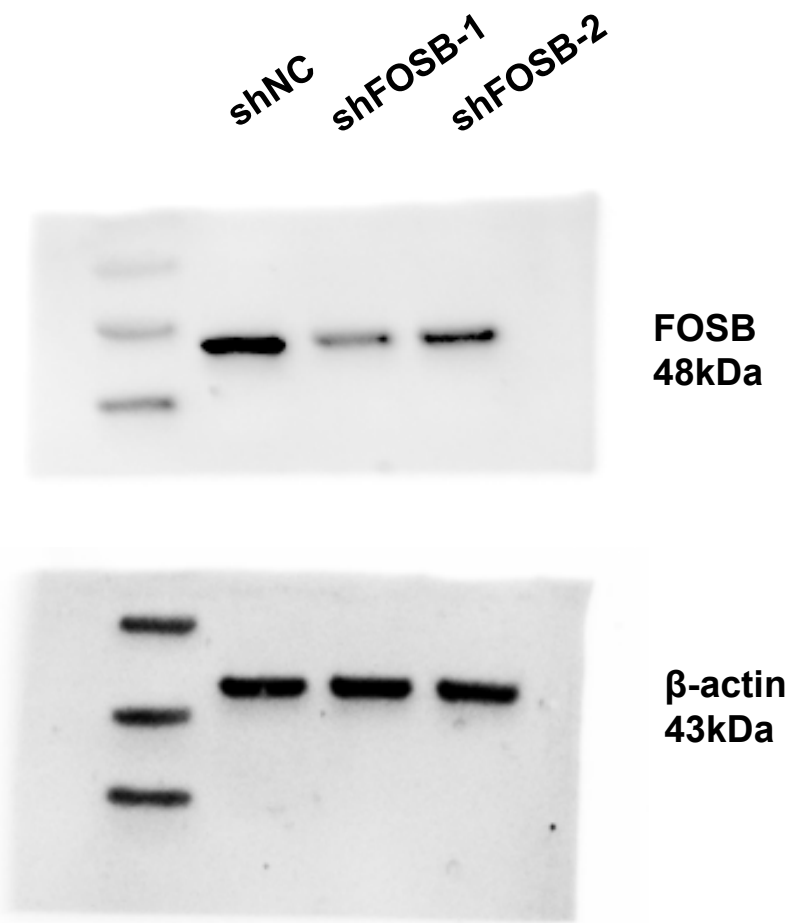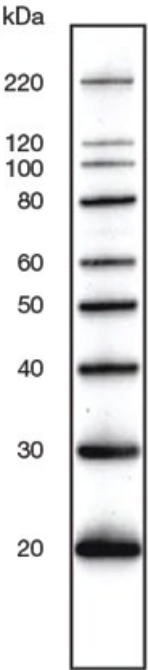

Fig 2c

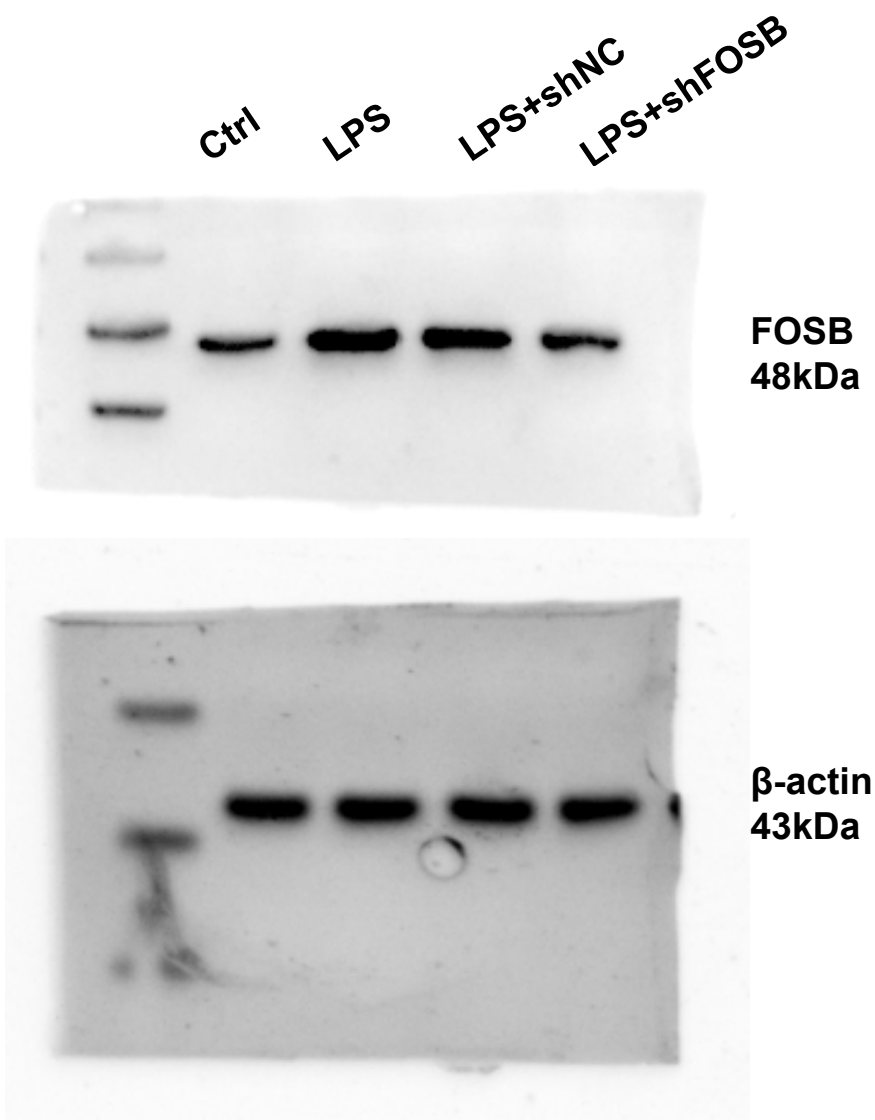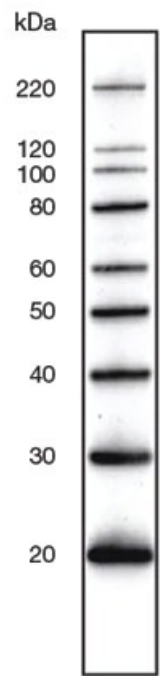

Fig 4f

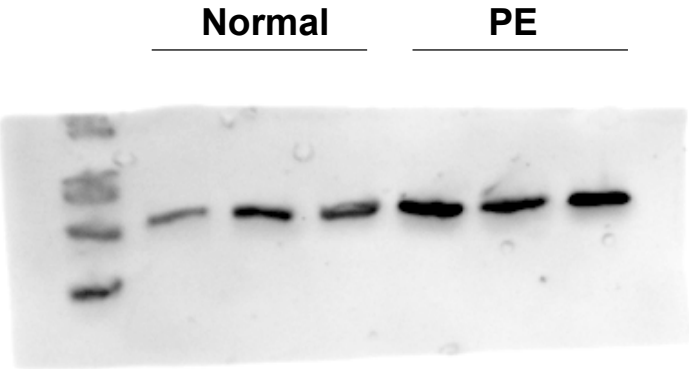

RBM15  
105kDa

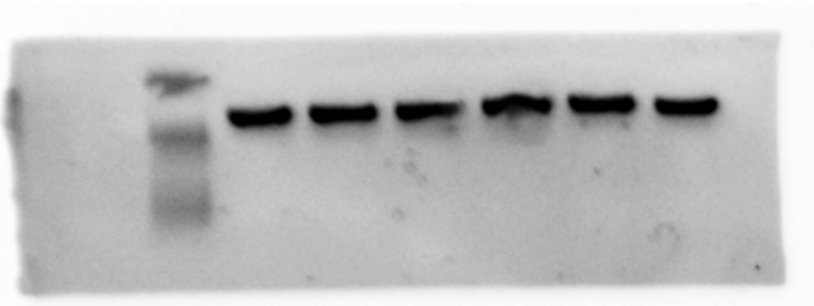

$\beta$ -actin  
43kDa

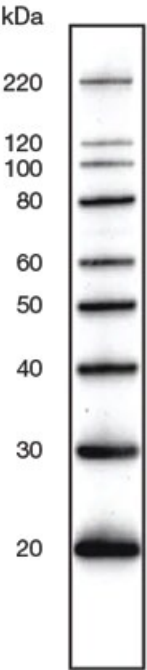

Fig 4g

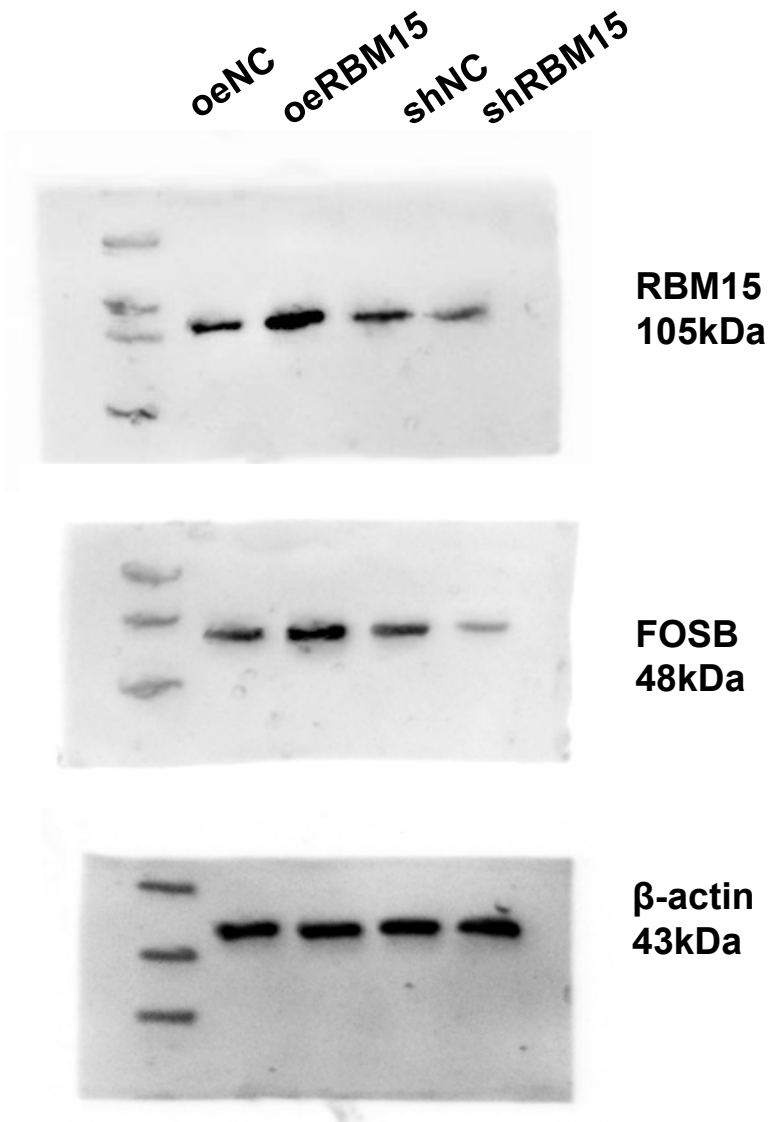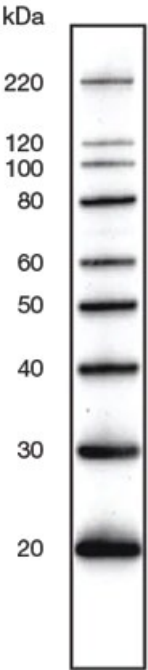

Fig 5c

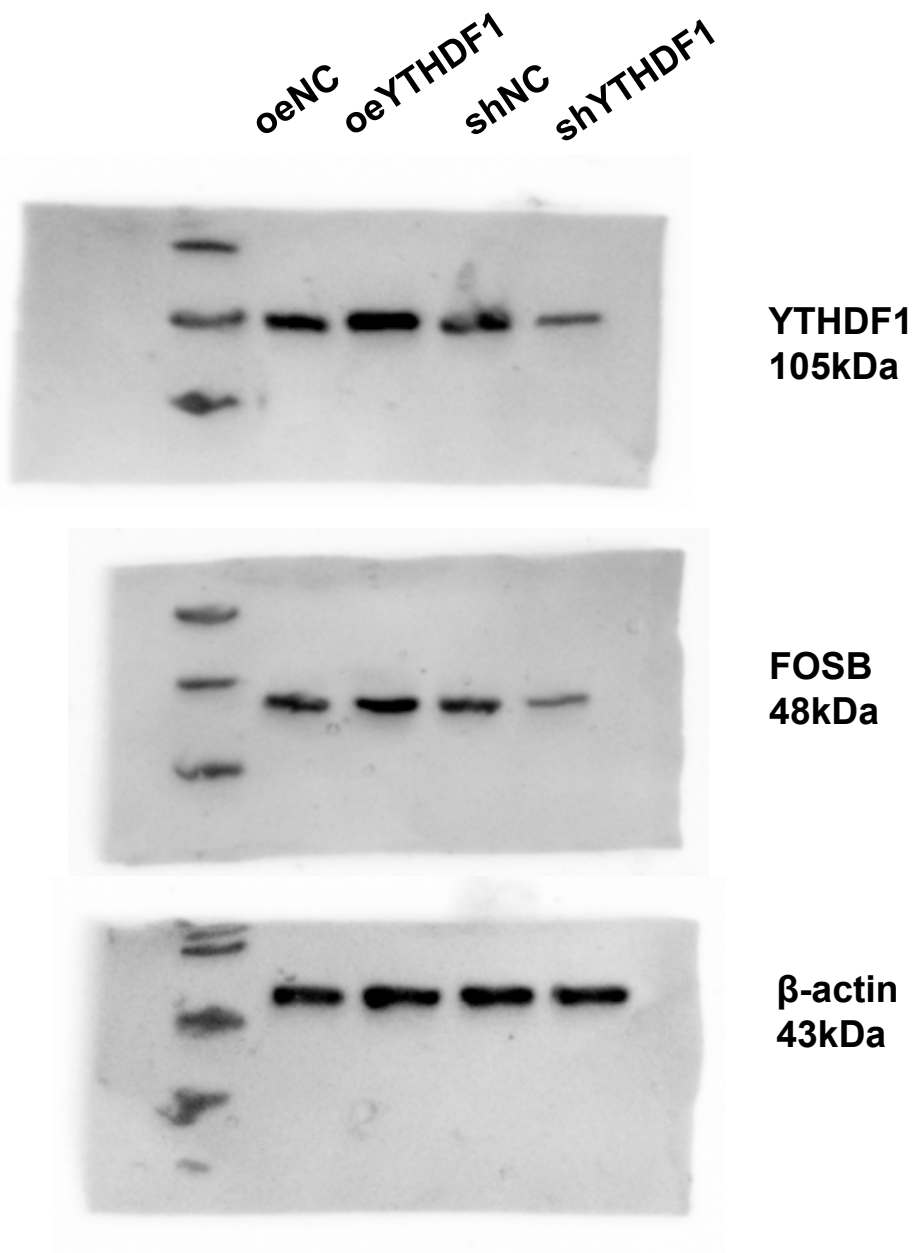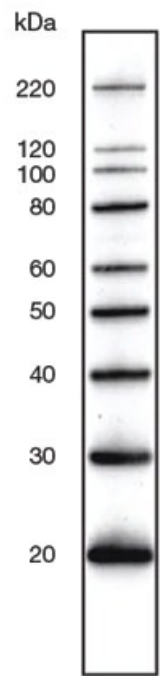

Fig 6a

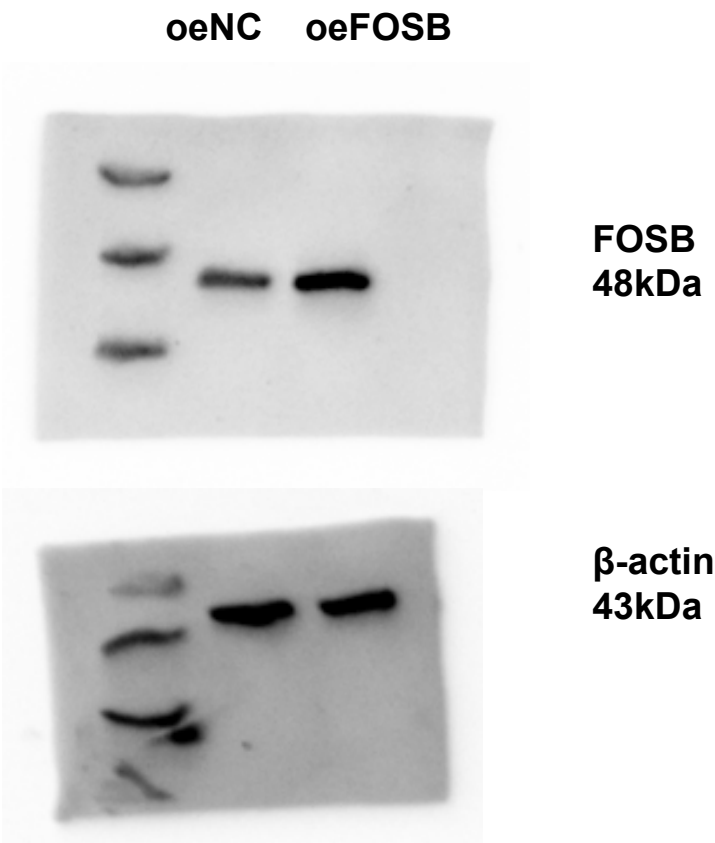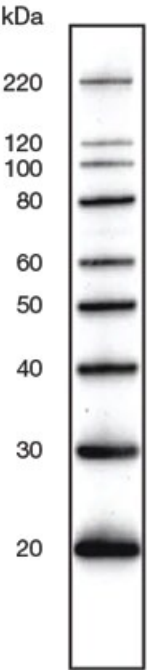

Fig 6b

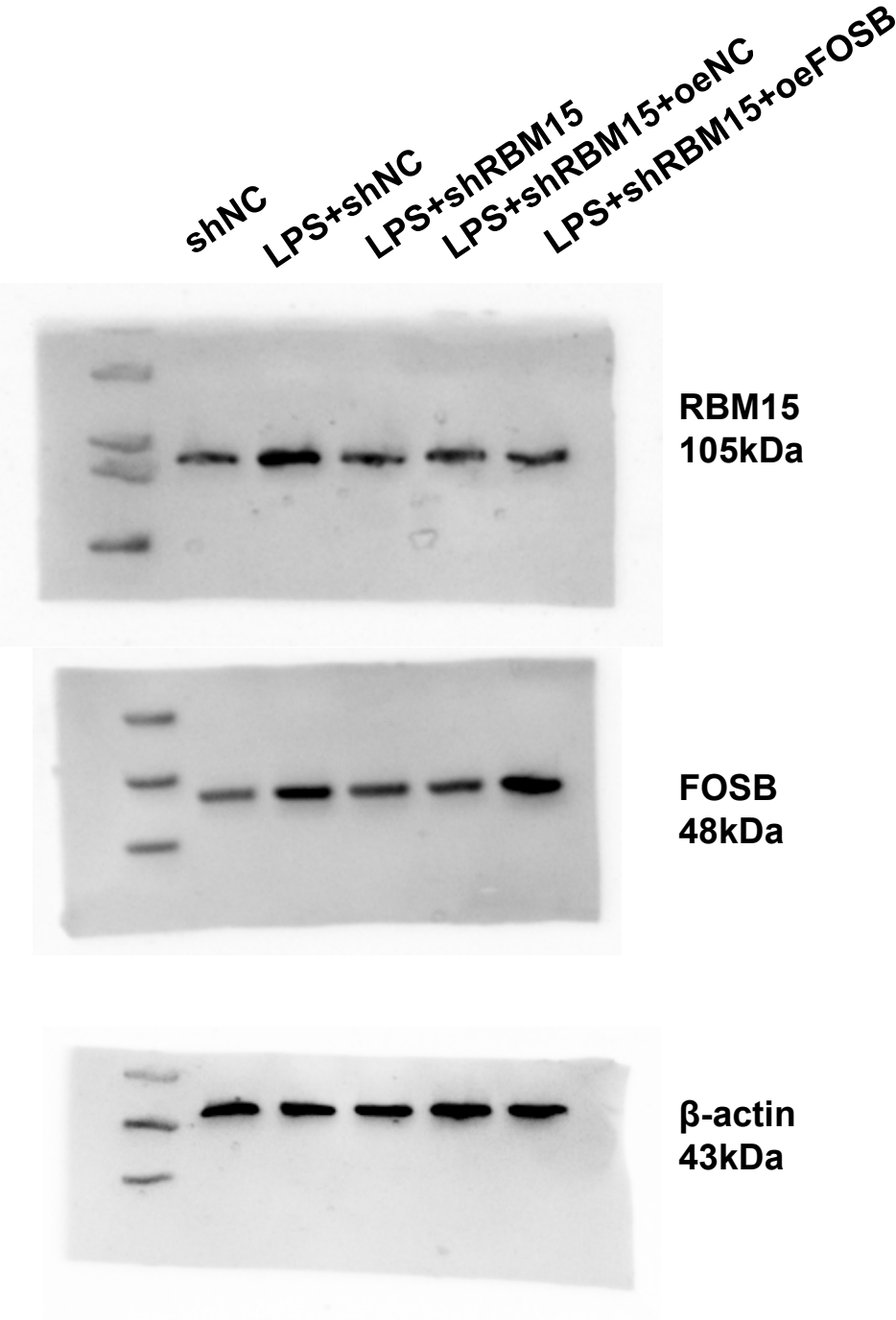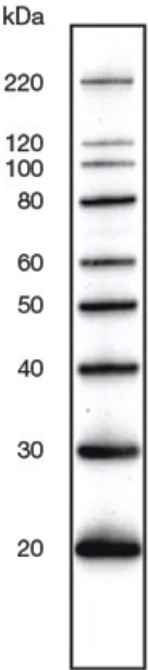

Supplement: Supplementary file 1 — Supplementary Material 1. [file 41065_2025_592_MOESM1_ESM.pdf]
