# Supplementary material for: RBM15 mediates m6A methylation modification of FOSB mRNA to promote dysfunction of trophoblast cells-a potential link to preeclampsia
Source: Hereditas. 2025 Nov 21;162:241. doi: 10.1186/s41065-025-00592-4 (PMC12752175; doi:10.1186/s41065-025-00592-4)
Supplement: Supplementary file 2 — Supplementary Material 2. [file 41065_2025_592_MOESM2_ESM.pptx]

## Slide 1
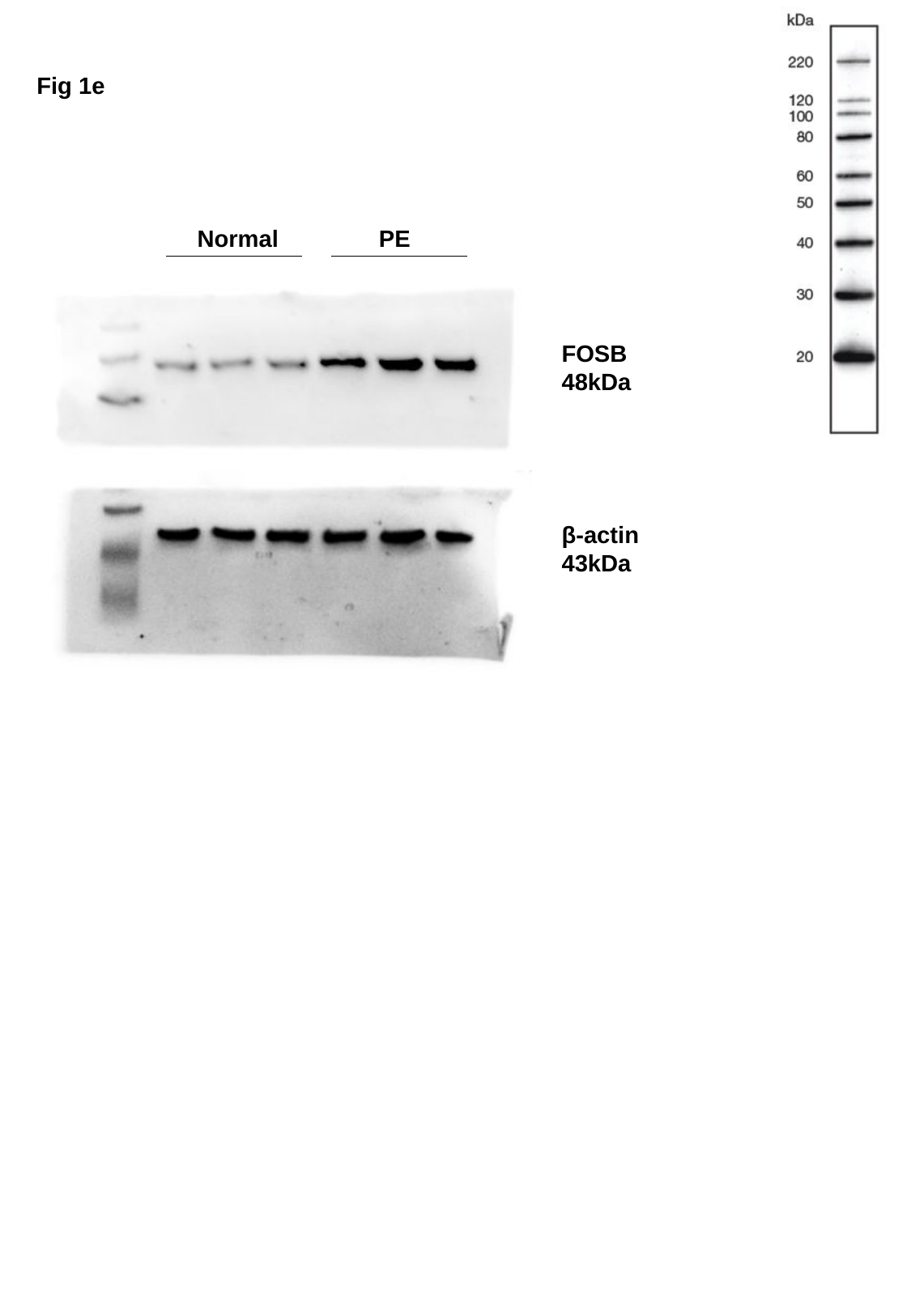

Fig 1e
Normal
PE
FOSB
48kDa
β-actin 43kDa

## Slide 2
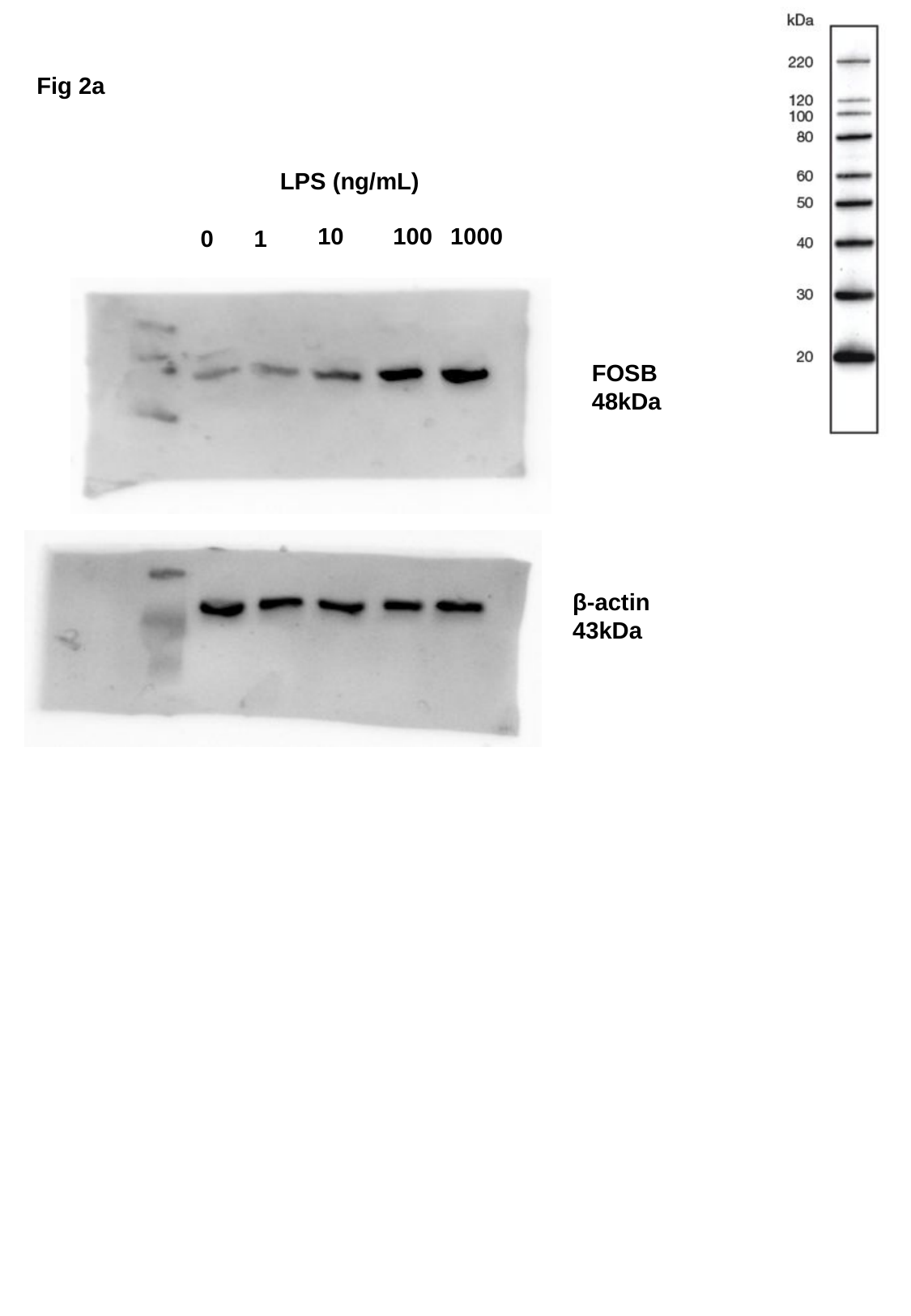

Fig 2a
LPS (ng/mL)
10
100
1000
0
1
FOSB
48kDa
β-actin 43kDa

## Slide 3
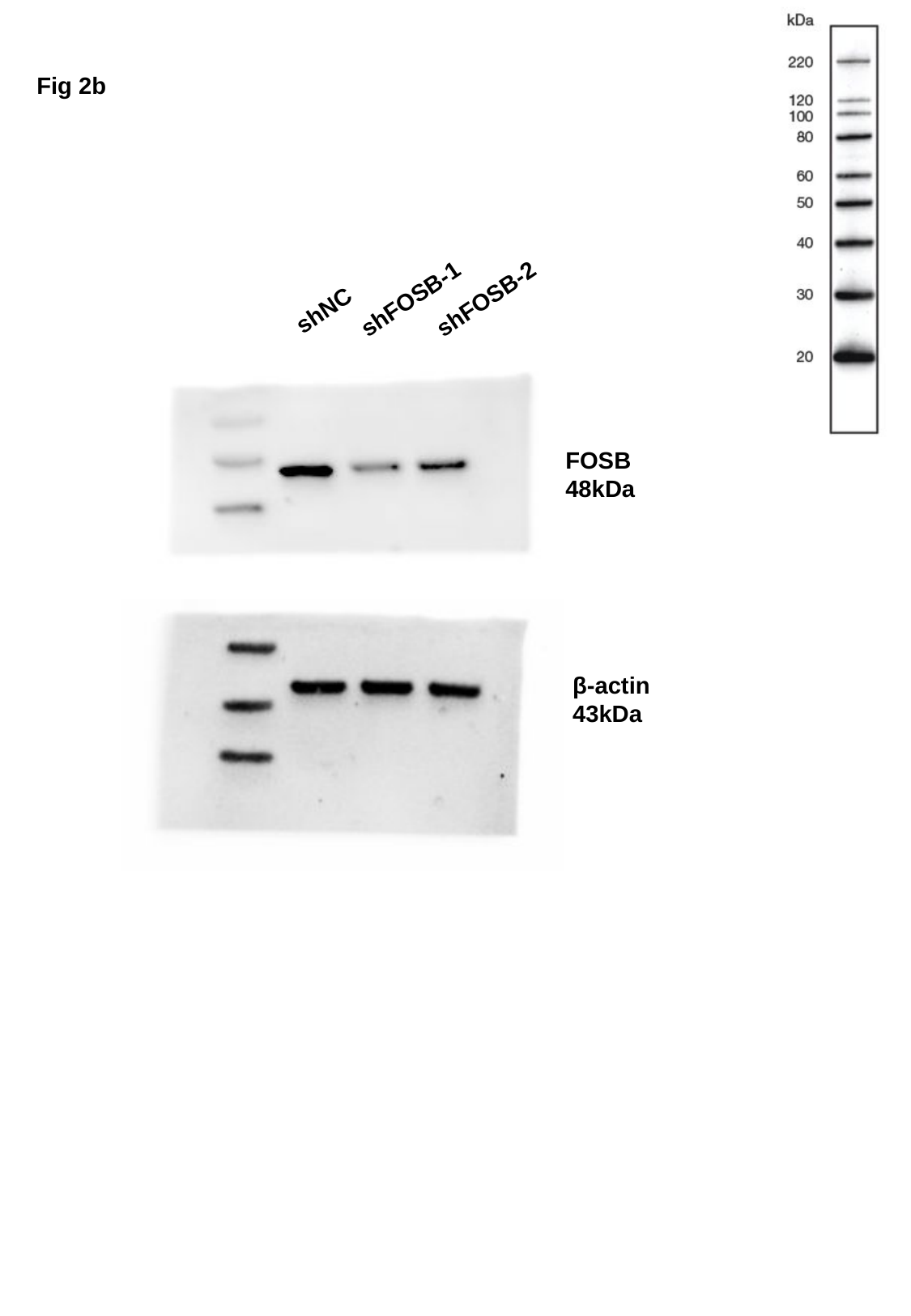

Fig 2b
shFOSB-1
shFOSB-2
shNC
FOSB
48kDa
β-actin 43kDa

## Slide 4
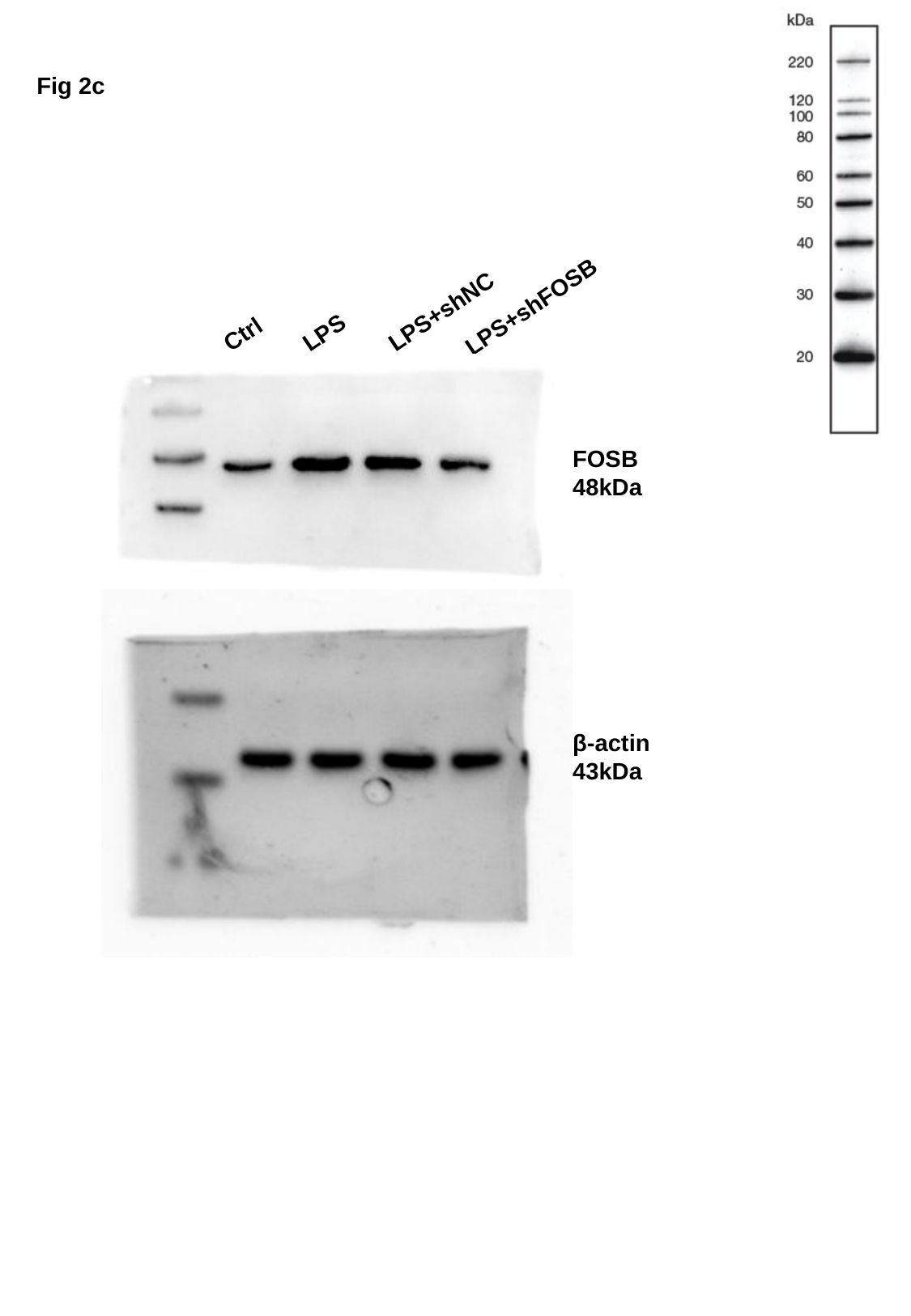

Fig 2c
LPS+shFOSB
LPS+shNC
Ctrl
LPS
FOSB
48kDa
β-actin 43kDa

## Slide 5
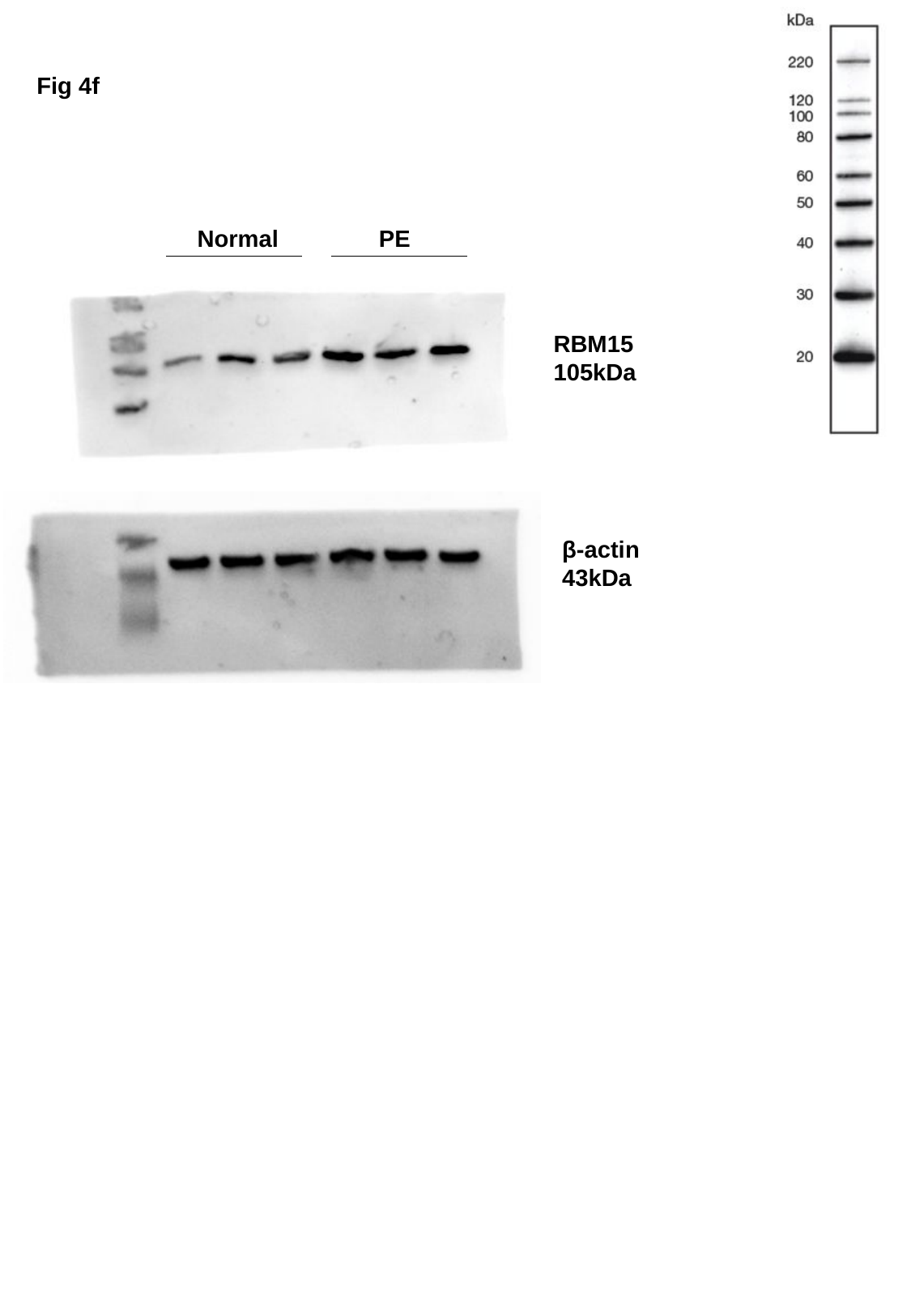

Fig 4f
Normal
PE
RBM15
105kDa
β-actin 43kDa

## Slide 6
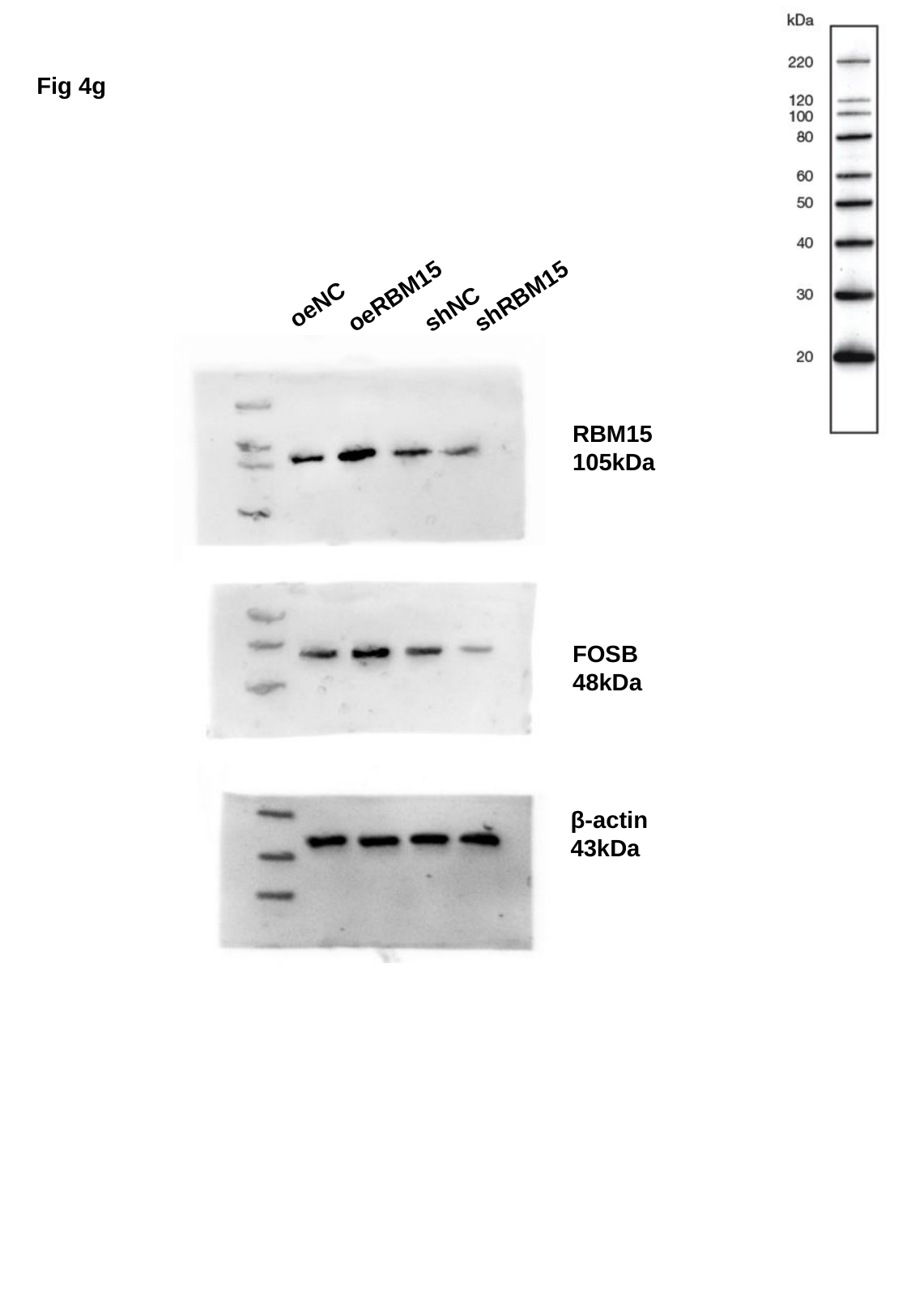

Fig 4g
shRBM15
shNC
oeNC
oeRBM15
RBM15
105kDa
FOSB
48kDa
β-actin 43kDa

## Slide 7
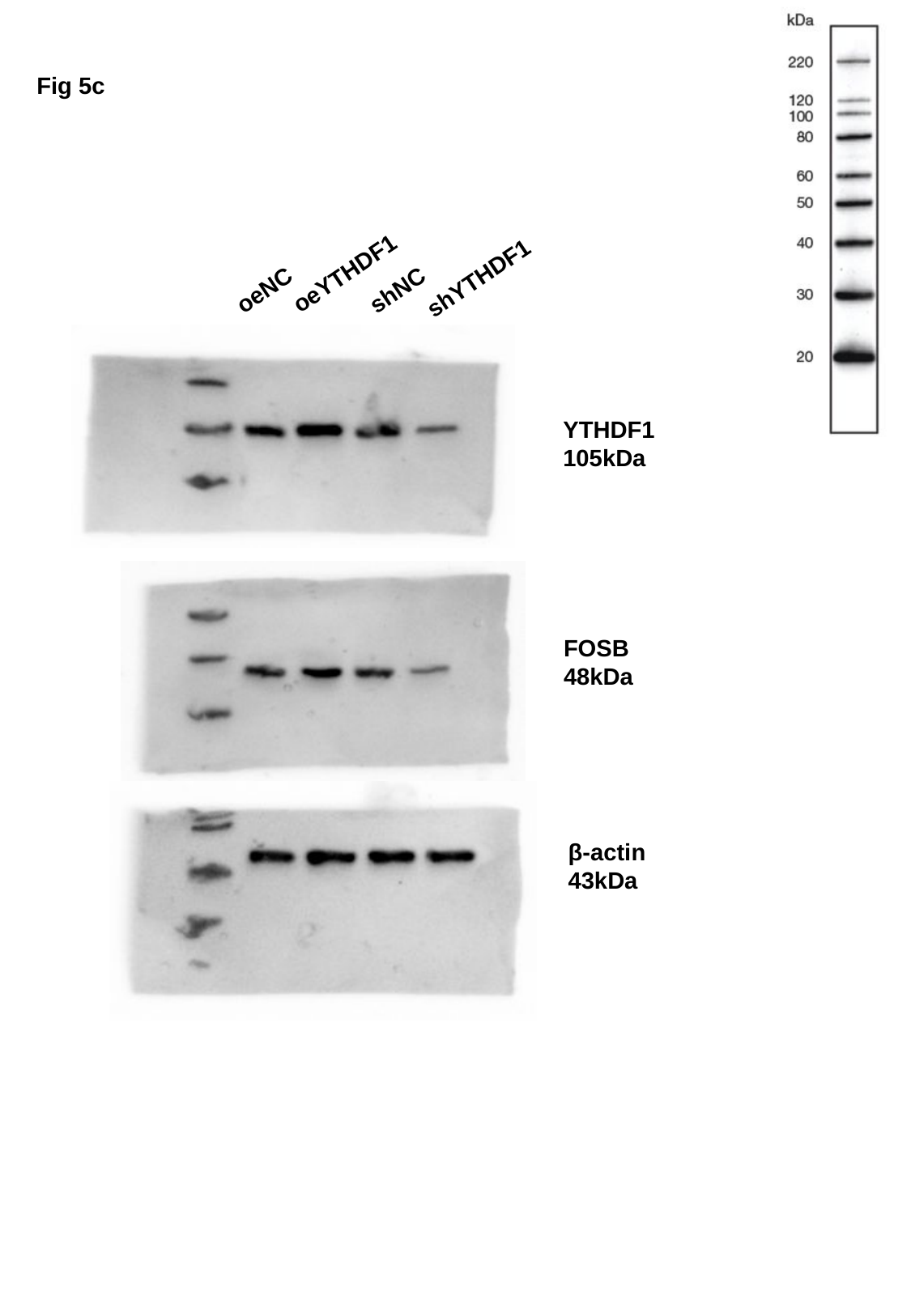

Fig 5c
shYTHDF1
shNC
oeYTHDF1
oeNC
YTHDF1
105kDa
FOSB
48kDa
β-actin 43kDa

## Slide 8
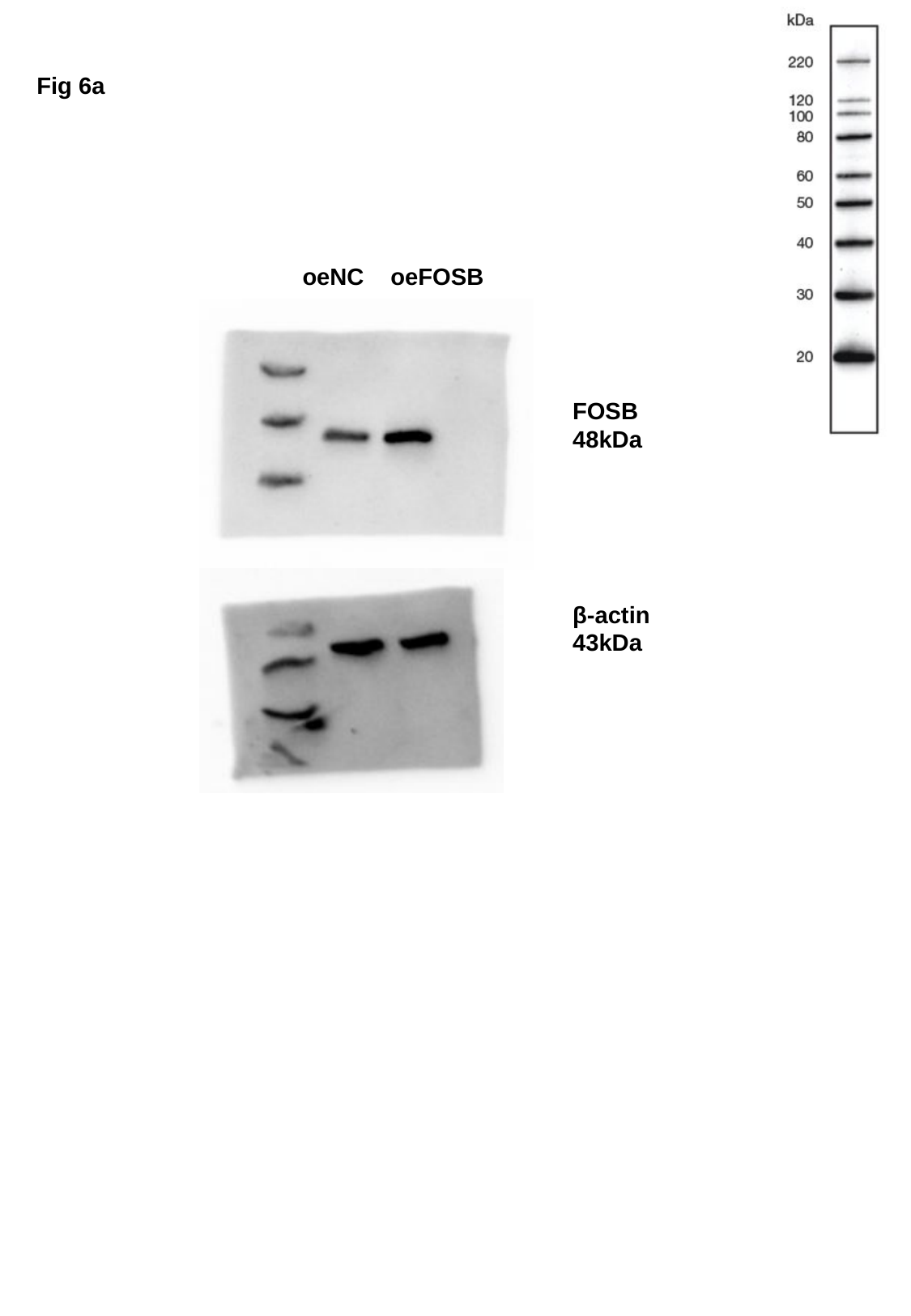

Fig 6a
oeNC
oeFOSB
FOSB
48kDa
β-actin 43kDa

## Slide 9
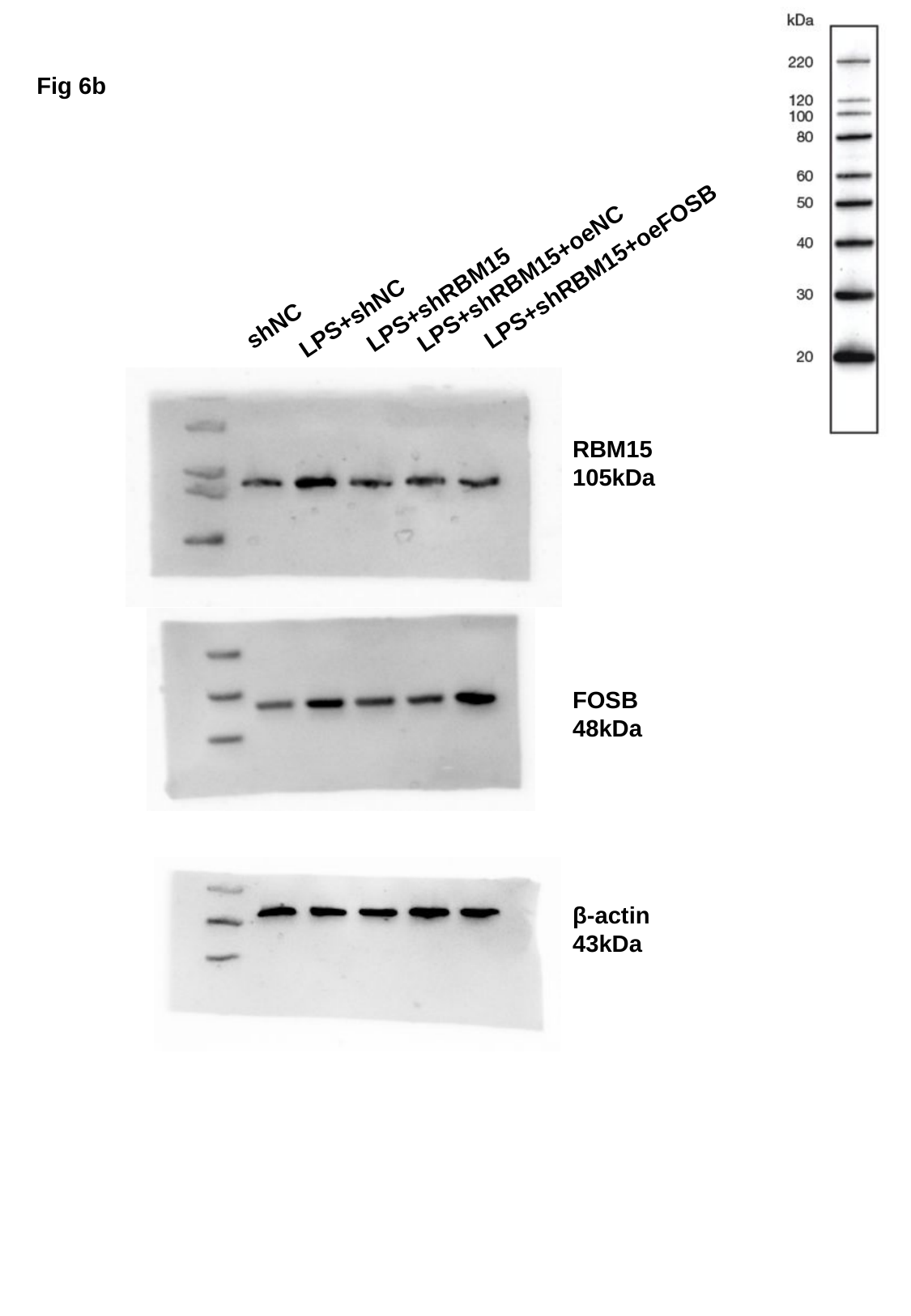

Fig 6b
LPS+shRBM15+oeFOSB
LPS+shRBM15+oeNC
LPS+shRBM15
shNC
LPS+shNC
RBM15
105kDa
FOSB
48kDa
β-actin 43kDa
